# Carbon Nanofiber Arrays: A Novel Tool for Microdelivery of Biomolecules to Plants

**DOI:** 10.1371/journal.pone.0153621

**Published:** 2016-04-27

**Authors:** Sandra M. Davern, Timothy E. McKnight, Robert F. Standaert, Jennifer L. Morrell-Falvey, Elena D. Shpak, Udaya C. Kalluri, Joanna Jelenska, Jean T. Greenberg, Saed Mirzadeh

**Affiliations:** 1 Biosciences Division, Oak Ridge National Laboratory, Oak Ridge, Tennessee, United States of America; 2 Electrical & Electronics Systems Research Division, Oak Ridge National Laboratory, Oak Ridge, Tennessee, United States of America; 3 Biology & Soft Matter Division, Oak Ridge National Laboratory, Oak Ridge, Tennessee, United States of America; 4 Nuclear Security & Isotope Technology Division, Oak Ridge National Laboratory, Oak Ridge, Tennessee, United States of America; 5 Department of Biochemistry and Cellular & Molecular Biology, University of Tennessee, Knoxville, Tennessee, United States of America; 6 Department of Molecular Genetics and Cell Biology, The University of Chicago, Chicago, Illinois, United States of America; Texas A&M University, UNITED STATES

## Abstract

Effective methods for delivering bioprobes into the cells of intact plants are essential for investigating diverse biological processes. Increasing research on trees, such as *Populus* spp., for bioenergy applications is driving the need for techniques that work well with tree species. This report introduces vertically aligned carbon nanofiber (VACNF) arrays as a new tool for microdelivery of labeled molecules to *Populus* leaf tissue and whole plants. We demonstrated that VACNFs penetrate the leaf surface to deliver sub-microliter quantities of solution containing fluorescent or radiolabeled molecules into *Populus* leaf cells. Importantly, VACNFs proved to be gentler than abrasion with carborundum, a common way to introduce material into leaves. Unlike carborundum, VACNFs did not disrupt cell or tissue integrity, nor did they induce production of hydrogen peroxide, a typical wound response. We show that femtomole to picomole quantities of labeled molecules (fluorescent dyes, small proteins and dextran), ranging from 0.5–500 kDa, can be introduced by VACNFs, and we demonstrate the use of the approach to track delivered probes from their site of introduction on the leaf to distal plant regions. VACNF arrays thus offer an attractive microdelivery method for the introduction of biomolecules and other probes into trees and potentially other types of plants.

## Introduction

Evaluating the function of a wide range of molecules, from proteins and nucleic acids to hormones and micronutrients, is essential to deciphering their roles in plant physiology. Whether molecules exert their effects locally or over a long-distance has important implications for metabolic coordination and signal propagation within the plant, as well as our ability to understand them. Numerous methods are used to introduce labeled molecules into plants for functional characterization. Genetic methods are often used to introduce heterologous proteins, as well as to induce overexpression or silencing of selected genes. Stable transgenics can be prepared readily with some model plants, notably *Arabidopsis*, but can require considerable time and effort to prepare with other models, such as *Populus*, the first woody perennial plant to have its genome sequenced and a commonly used model for studies of vascular development, biomass production and stress responses [1]. Transient transformation, using for example biolistic bombardment or *Agrobacterium tumefaciens*-mediated gene delivery, is a useful alternative for these reasons [2–5].

A constraint shared by all genetic methods is that they are limited to expressible tags, such as green fluorescent protein. Physical methods, which lack this constraint, provide an important complement for introducing molecules that can be tracked and studied. These methods allow greater flexibility with respect to plant species, site of introduction, rate of introduction and, in particular, the types of probes and tags that can be introduced. For example, probes labeled with biotin, synthetic fluorophores and radiotracers can enable a wider variety of experiments. Fluorescent tags are an ideal way to visualize local cellular effects, whereas γ-emitting radioisotopes, such as ^125^I, are useful to track movement throughout the plant.

Among the commonly employed physical methods are root irrigation, foliar application, and pressure infiltration. These methods are routinely employed to facilitate uptake of a variety of molecules by plants for subsequent study [5–9, 10]. However, they are limited by the natural permeability barriers around and within plants. The leaf epidermis presents a triple barrier of the cuticle, the cell wall and the cell membrane; hence it resists penetration by many molecules, including proteins and nucleic acids. Entry of exogenous materials into leaves via stomata is less restrictive and allows delivery of particles as large as bacteria into the apoplast; the process can be accelerated by pressure infiltration, at the expense of some localized wounding. Roots are selective in their uptake of cargoes and are of little value for delivering large biomolecules such as proteins. While localized adsorption and uptake of proteins into root cells can occur, systemic transport to the extent that it occurs is believed to be the result of root damage or stress, and some proteins are degraded either prior to or after uptake [11–14].

Tissue and vasculature can be exposed for direct delivery of probes by more aggressive methods that breach the natural barriers, such as scission of a petiole, nicking, puncturing or crushing of tissue and abrasion or peeling of the leaf epidermal surface [15–17]. For example, abrasion with carborundum is often used to introduce DNA [18], *Agrobacterium* [19, 20] and viruses [21, 22] to plants. These approaches are simple and widely used but have the common drawback of wounding the plant. More specialized methods have also been developed for particular applications. One interesting example is microinjection through insect stylets, which allows microdelivery into single sieve elements of the phloem [23, 24]. Because all of these methods have limitations or shortcomings, new methods are actively pursued. For example, a method to perforate the cuticle of citrus leaves with laser light for enhanced foliar uptake was recently reported [25].

**Fig 1:**
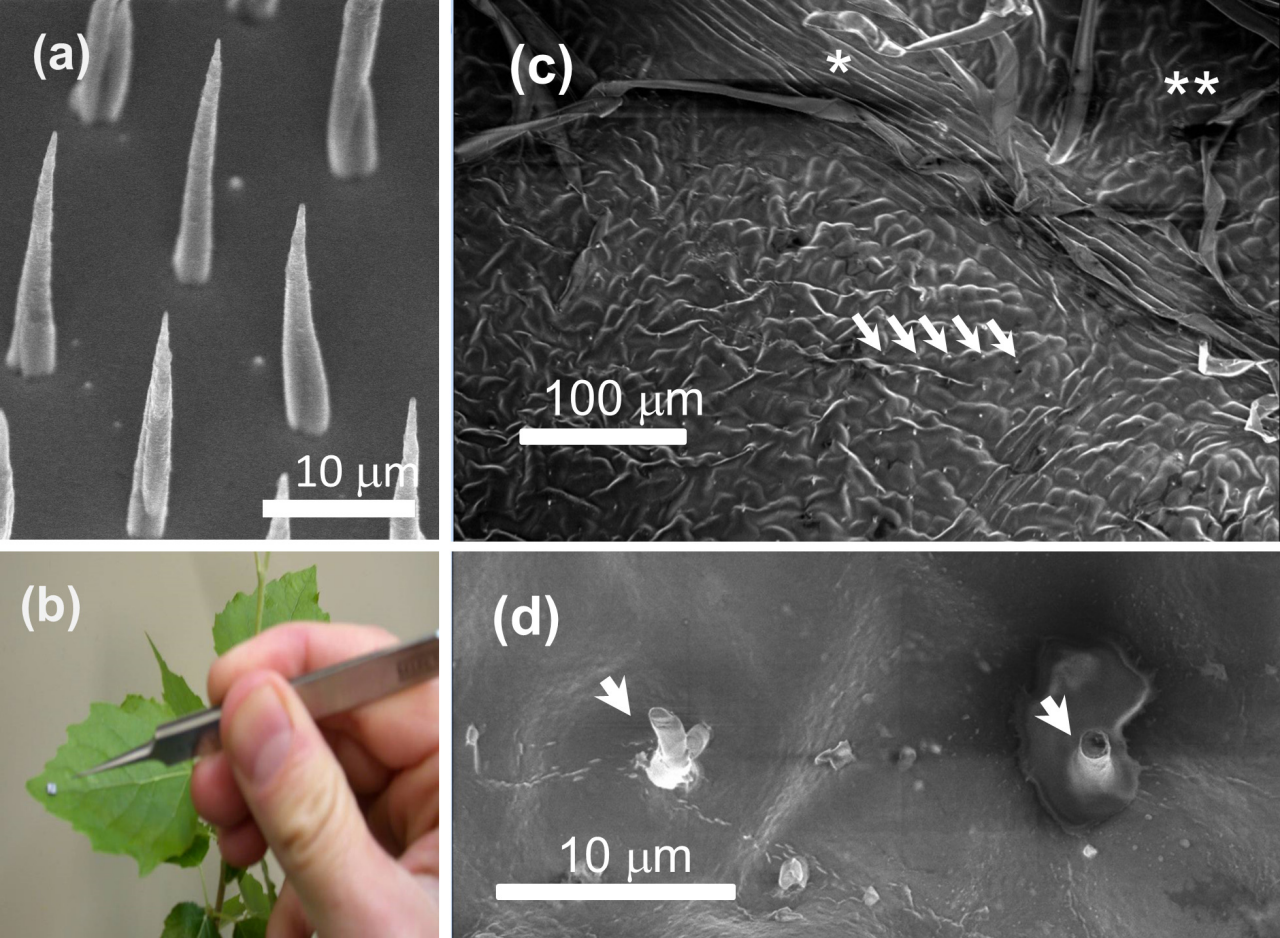
Vertically-aligned carbon nanofiber arrays provide densely clustered, microscopic spikes that penetrate leaf tissue. (a) Scanning electron micrograph (SEM) of a small region of a VACNF array chip. Nanofibers are arrayed across this chip at a 10 μm pitch. Individual nanofiber heights were 20–25 μm. In the image, the chip is tilted 30° from perpendicular to the beam to show the aspect ratio of each nanofiber. (b) Application of a carbon nanofiber chip onto the adaxial surface of a *Populus* leaf. (c) SEM of a leaf impaled with a VACNF chip (20-μm pitch), showing fibers embedded in the epidermal cells of the adaxial surface as indicated by the arrows. The position of a minor vein adjacent to the site of impalement is marked by (*), and a region of unimpaled tissue is marked by (**). (d) A close-up view of the impalement site, showing individual carbon nanofibers that have broken off of the VACNF chip and remain embedded in the leaf epidermal cells, as indicated by arrows. The image was taken ~15 min after VACNF impalement. Excised leaf tissue was placed directly in the electron microscope, where it was dried *in vacuo* prior to imaging. Results shown are from single, typical experiments.

Vertically-aligned carbon nanofibers (VACNFs) are synthetic nanostructures similar in key dimensions (length, diameter and taper) to insect stylets [26], with individual fibers typically having tip diameters of ˂100 nm, base diameters of ~500 nm, and controllable lengths from a few μm to approximately 100 μm [27]. Unlike stylets, however, the tip of each fiber is closed (Fig 1A). Carbon nanofibers are physically robust, such that they can impale tissue, and they can be fabricated in defined patterns on a variety of solid substrates. These properties have been exploited in the development of VACNF arrays for a massively parallel, microinjection-like approach to delivering DNA or small, membrane-impermeant molecules into mouse myeloma and Chinese hamster ovary cells [28, 29].

Based on these precedents, it was anticipated that VACNFs might also provide an effective means of delivering exogenous molecules into plant tissue. Here, we report the successful use of VACNF arrays to deliver femtomole to picomole (10⁻^15^ to 10⁻^12^ mol) quantities of labeled molecules from 2-μL droplets into the epidermis and palisade layer of *Populus* leaves without compromising epidermal cell integrity or inducing a detectable wound response. The technique was demonstrated using a set of molecules of diverse sizes and types, including the fluorescent dye Lucifer Yellow CH (LYCH; relative molecular mass *M*_r_ = 0.5 kDa, hydrodynamic radius *R*_h_ = 0.68 nm [30]), small proteins (*M*_r_ = 15–30 kDa, *R*_h_ = 2.0–2.5 nm [31]) and high molecular weight dextran (*M*_r_ = 500 kDa, *R*_h_ = 15.9 nm [32]). Using fluorescent and radioactive labels, we monitored movement of proteins from the site of delivery and found the smaller molecules to be mobile, with proteins moving throughout the plant, whereas the large dextran was restricted to the site of delivery.

## Results

### VACNFs penetrate the epidermis of *Populus* leaves without causing detectable tissue damage or wound response

For microdelivery to plants, VACNFs were grown 20–25 μm long with a 1-μm base diameter tapering to a sharp tip (<100 nm diameter). The fibers were thus long enough to penetrate into the leaf epidermis but much narrower than a typical plant cell. SEM was used to verify that a uniform array of fibers with the desired dimensions had grown (Fig 1A).

To test the effectiveness of the fiber arrays at penetrating plant tissue, mock treatments of *Populus* leaves were performed with small (2 × 2 mm), dry chips (Fig 1B). A gentle tap on the chip with forceps was sufficient to drive the fibers into plant tissue. SEM images of treated leaves taken 15 min after chip removal showed multiple fibers had broken off and remained embedded within leaf epidermal cells at a density of about one fiber per cell (Fig 1C) when using VACNF arrays with a 20-μm pitch. Impaled epidermal cells were similar in appearance to adjacent cells without fibers (Fig 1C), and the overall morphology of the impaled tissue was indistinguishable from that of adjacent regions (Fig 1C). Close inspection revealed some material, perhaps exudate, around the stubs of penetrant nanofibers, as seen on the right in Fig 1D. It is unknown whether this material was released from cells during the impalement process or is an artifact of SEM imaging, during which the impaled tissue was placed under vacuum and desiccated.

**Fig 2:**
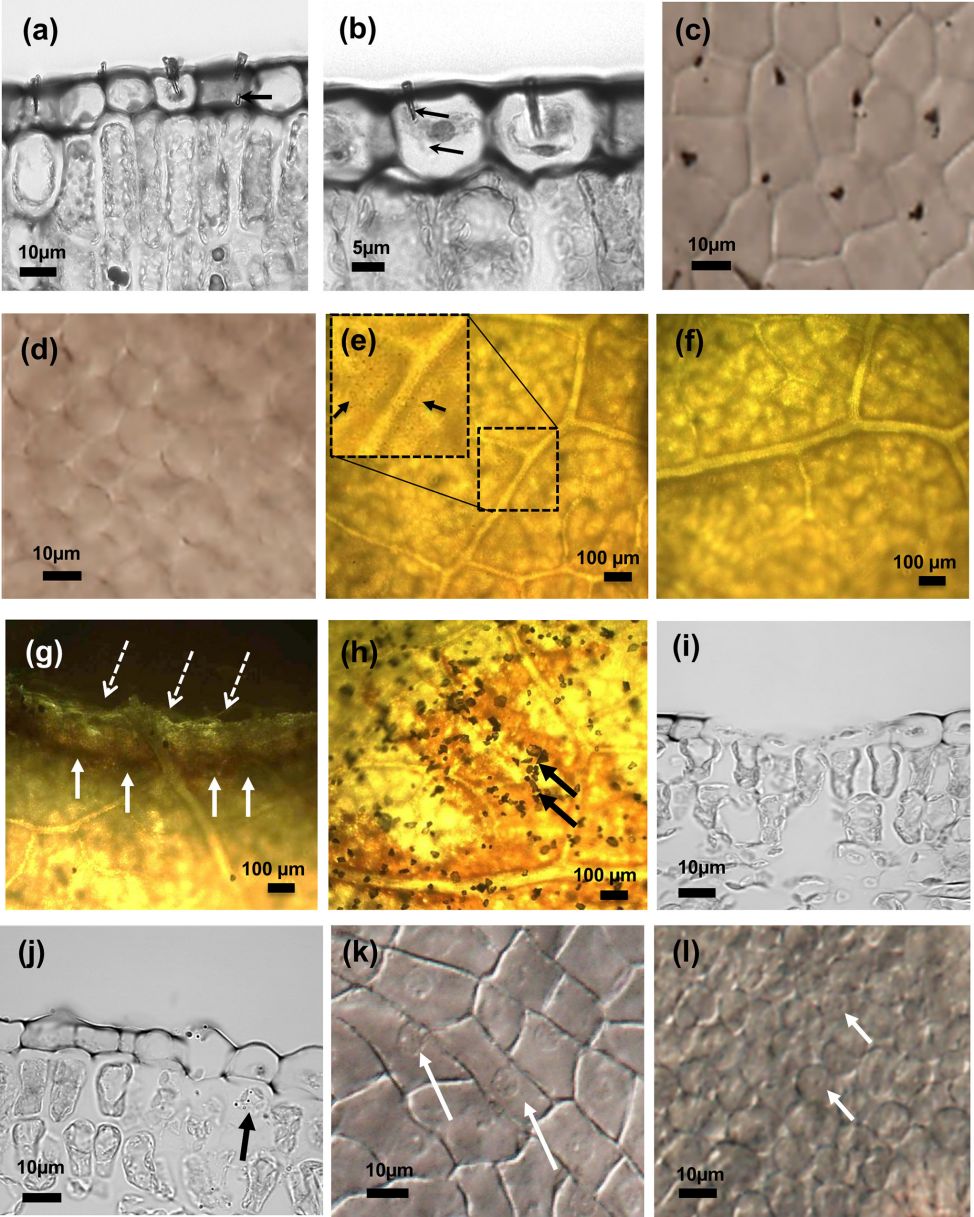
VACNFs penetrate *Populus* epidermis without damaging cells. (a) and (b) Micrographs of transverse sections through a *Populus* leaf showing individual nanofibers penetrating the cuticle and epidermis. The arrow in (a) shows a nanofiber reaching the base of an epidermal cell without penetrating the underlying palisade cell. Arrows in (b) indicate a nanofiber traversing the cytoplasm of the cell. (c) DIC micrograph of a leaf epidermis showing that carbon nanofiber impalement occurs in a grid pattern, similar to the original chip. (d) DIC micrograph of the same leaf depicted in (c) with the focal plane moved into the underlying palisade layer showing the absence of over-penetrant fibers. Results are representative of leaves from 2 separate plants. (e) and (f) Bright-field micrographs of *Populus* leaves after staining with DAB to detect H_2_O_2_ production. Leaves penetrated by carbon nanofibers (e) show no DAB staining and are similar to untreated areas of the leaf (f). The boxed inset in (e) shows a magnified image of the nanofiber-treated area, with black arrows indicating the location of carbon nanofibers in this image. (g) and (h) Leaves wounded with a cork borer (g) or abraded with carborundum (h) show areas stained dark brown by DAB deposition in reaction to H_2_O_2_ produced in the wound response. Black arrows in (h) indicate carborundum powder remaining on the leaf. Dashed arrows in (g) point to the cut edge of the leaf and solid arrows indicate staining with DAB. Similar results were obtained with leaves from 3 separate plants. (i) and (j) Transverse sections of a *Populus* leaf after carborundum abrasion, showing areas of (i) severe and (j) mild epidermal damage. The black arrow in (j) points to grit particles within the palisade layer. (k) DIC micrograph of a leaf epidermis after carborundum treatment showing abraded epidermal cells (denoted by white arrows). (l) DIC micrograph of the same leaf depicted in (k) with the focal plane moved into the underlying palisade layer, showing the presence of embedded grit particles (denoted by white arrows). Images (a), (b), (i) and (j) were obtained from thin sections of fixed (formalin), embedded (paraffin) and stained (toluidine blue) tissue; images (c), (d), (k) and (l) were obtained from fixed (ethanol-acetic acid) and cleared (chloral hydrate) tissue; images in (e)–(h) were obtained from stained (DAB) and decolorized (boiling ethanol) tissue. Details are provided in *Materials and Methods*.

Examination of transverse sections of VACNF-impaled *Populus* leaves by optical microscopy revealed an intact epidermis with embedded nanofibers. In 188 impaled cells examined, the fibers had penetrated through the cuticle and the epidermal cell wall into the cytoplasm without causing the cells to collapse or burst (Fig 2A and 2B). In seven instances, fibers extended to the base of epidermal cells, as indicated by the arrow in Fig 2A, but none were observed to breach the underlying palisade layer. DIC micrographs of the leaf surface illustrated the regularity of VACNF impalement into the epidermis as well as the preservation of overall tissue morphology (Fig 2C). The underlying palisade layer showed no evidence of fibers (Fig 2D). Production of H_2_O_2_, which would indicate a wound response, was not detected (Fig 2E), and treated tissue looked similar to untreated tissue (Fig 2F). In contrast, cutting leaves with a cork-borer or gently rubbing the leaf surface with carborundum elicited H_2_O_2_ production (Fig 2G and 2H) and caused damage that was clearly visible in transverse sections (Fig 2I and 2J). In the more badly damaged areas, carborundum treatment caused removal of the cuticle and epidermal cells, (Fig 2I). In addition, grit remained within the epidermis and was also found within the palisade layer (Fig 2J). DIC microscopy revealed epidermal abrasion (Fig 2K) and confirmed the presence of grit in the underlying palisade layer (Fig 2L) when carborundum powder was used. Thus, VACNF array penetration is more tissue-selective and less injurious than abrasion with carborundum.

### VACNF arrays deliver cargo molecules directly inside impaled cells and into the apoplastic space

**Fig 3:**
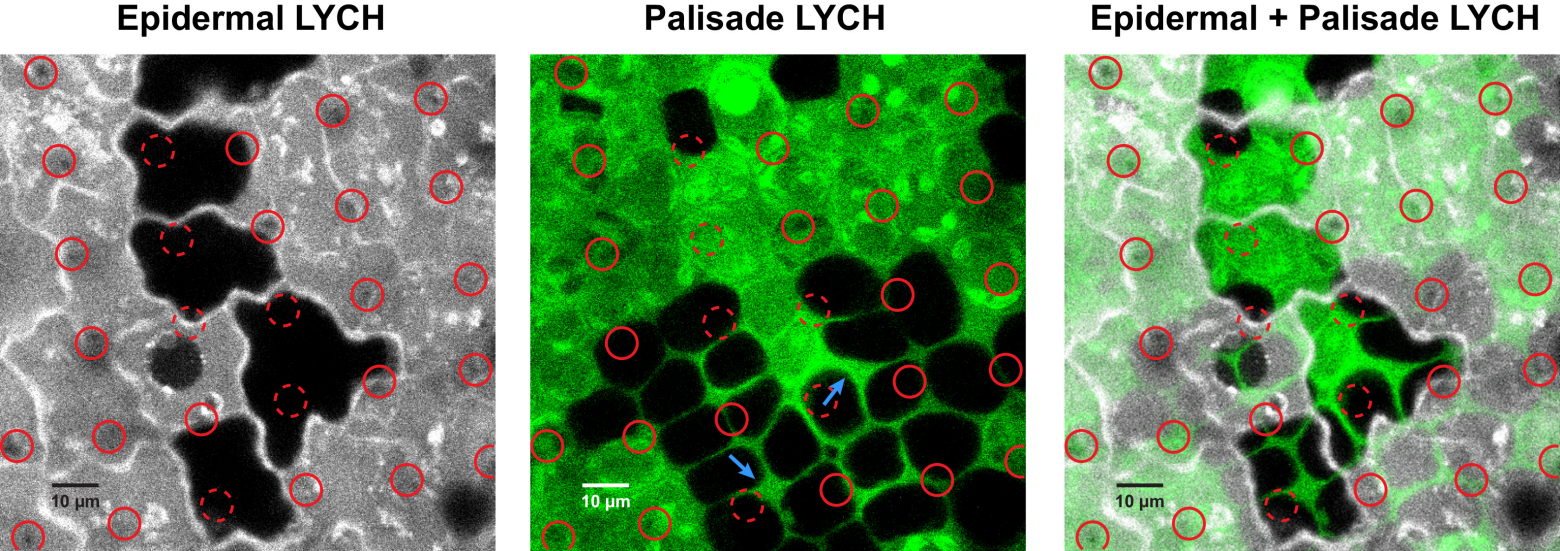
VACNFs Deliver LYCH to symplast and apoplast in *Populus* leaf tissue. LYCH (1 mM) was applied to the adaxial surface of a *Populus* leaf, and a VACNF array was placed on top and pressed to penetrate the leaf surface. Uptake of the solution was allowed to proceed for 5 minutes, after which the leaf was removed from the plant, the chip area was excised with a scalpel and the chip was removed. The leaf tissue was gently washed to remove surface LYCH, sealed under a cover glass and imaged immediately using confocal laser scanning microscopy. Solid red circles indicate locations of observed nanofibers, whereas dashed red circles indicate locations where nanofibers would be expected but were not definitively observed. (left) LYCH fluorescence signal from the epidermal layer (false-colored white). (center) LYCH fluorescence signal from the palisade layer, 9 μm deeper in the leaf (false-colored green). Arrows indicate LYCH in the apoplast of the palisade layer. (right) Superposition of the left and center images.

Delivery experiments were performed with a small (0.5 kDa) membrane-impermeant dye, LYCH, which is frequently used to trace symplastic movement paths in plants [30, 33–37]. LYCH was delivered via VACNF into a *Populus* leaf, and after 5 min, the chip was removed and the leaf observed by confocal laser scanning microscopy (Fig 3). The dye was apparent in the cytoplasm of impaled epidermal cells and throughout the surrounding apoplast, which it could have reached directly, i.e., through impalement between cells, or indirectly, i.e., through diffusion out of cells. Examination of the underlying tissue showed that LYCH was also present in palisade cells and in the apoplast, despite lack of detectable penetration of fibers into the palisade layer. In contrast to these observations, no detectable uptake of LYCH into cells occurred with mock deliveries performed with smooth chips, nor did the dye enter via cut leaf edges during processing (S1 Fig). The uptake of LYCH only after treatment with the VACNF array indicates that nanofiber penetration results in permeablization of the leaf tissue to the dye.

**Fig 4:**
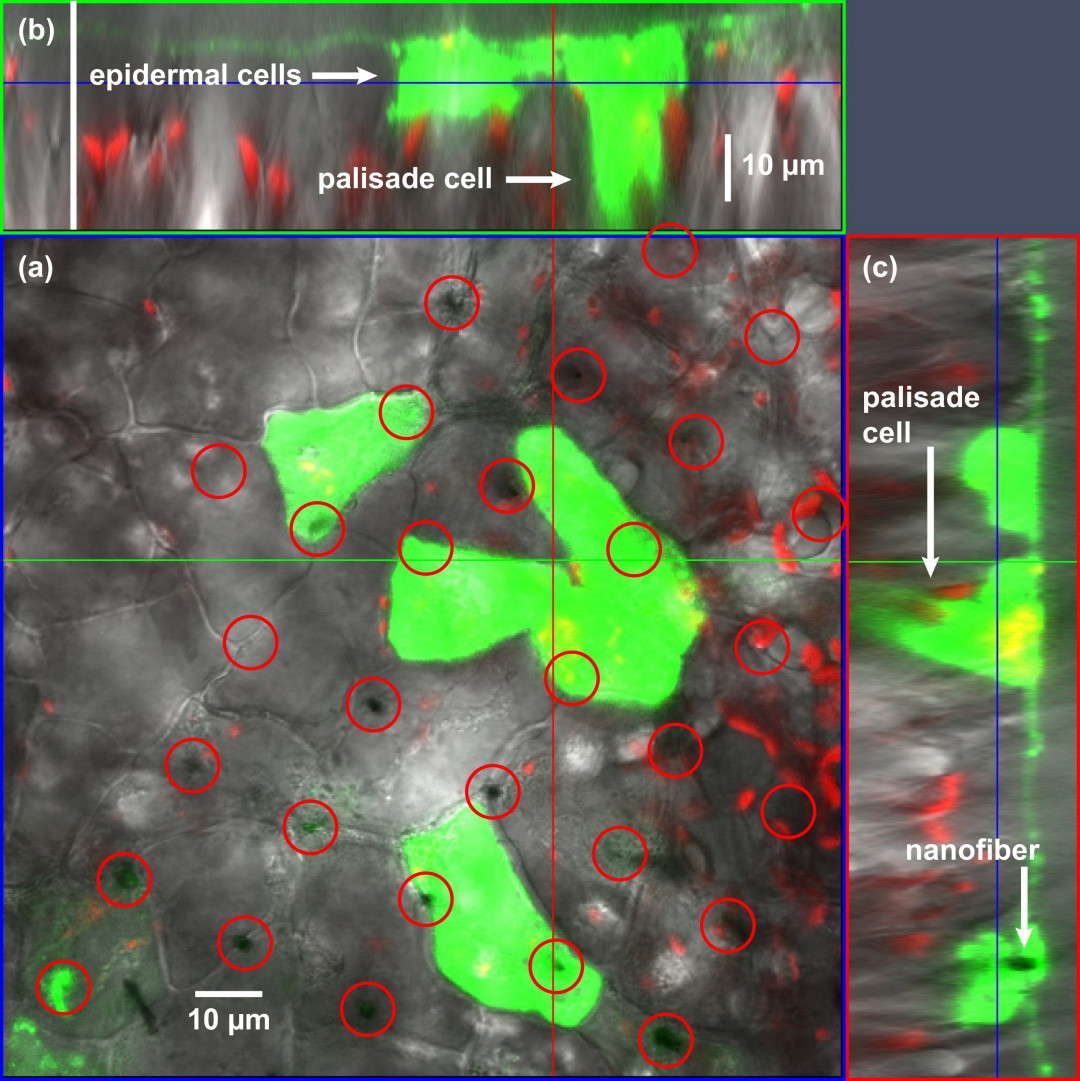
VACNFs deliver 500-kDa FITC-Dextran into epidermal and palisade cells in *Populus* leaf tissue. FITC-dexran (1 mM) was applied to the adaxial surface of a *Populus* leaf, and a VACNF array was placed on top and pressed to penetrate the leaf surface. Uptake of the solution was allowed to proceed for 5 minutes, after which the leaf was removed from the plant, the chip area was excised with a scalpel and the chip was removed. The leaf tissue was gently washed to remove surface FITC-dextran, sealed under a cover glass and imaged immediately using confocal laser scanning microscopy. Shown is the FITC fluorescence signal (false colored green), merged with the chlorophyll fluorescence signal (false colored red) from chloroplasts and the transmitted light signal (gray scale) for anatomical reference. (a) Shows the adaxial epidermal layer (x-y plane), where several cells are filled with FITC-Dextran. Red circles denote the locations of resident nanofibers. (b) and (c) Show orthogonal views (x-z and y-z planes, respectively) at locations indicated by the horizontal (x, green) and vertical (y, red) lines in (a). The blue lines in (b) and (c) indicate the image plane of (a). Arrows indicate the location of FITC-dextran-filled epidermal and palisade cells, as well as the location of a resident nanofiber (c). All filled palisade cells were directly beneath filled epidermal cells, and lateral migration of the dye within the palisade layer was not observed. Similar results were obtained for 3 separate experiments.

As LYCH is symplastically mobile [38–40], it was not clear whether it reached palisade cells by symplastic transport or by unseen nanofiber impalement. Therefore, we repeated the experiment using FITC-labeled, 500-kDa dextran (*R*_h_ = 15.9 nm), which is above the size-exclusion limit for symplastic transport via plasmodesmata [41–47]. This limit is considered for dextran to be ~3.7 kDa (*R*_h_ = 0.73 nm) or 9.4–17.2 kDa (*R*_h_ = 2.4–3.1 nm), depending on circumstances [44]. For globular proteins, the range in *R*_h_ of 2.4–3.1 nm corresponds to relative molar masses of ~30–70 kDa, consistent with the observation that green fluorescent protein (GFP, *M*_r_ = 27 kDa) is freely mobile in *Arabidopsis* embryos, whereas dimerized and trimerized forms of the protein have increasingly restricted movement [48, 49]. As was expected on the basis of these limits, the large dextran accumulated within impaled epidermal cells and in some instances was seen in underlying palisade cells (Fig 4) but did not migrate from the site of delivery. Nanofibers were not visible within palisade cells, so it appears that delivery occurred through transient nanofiber penetration during the initial pressing of the chip into the leaf. Impalement between cells was also evident, but in contrast to LYCH, FITC-dextran did not spread discernibly in the apoplast. The lack of movement of dextran could result from several factors, including the 20-fold lower rate of diffusion expected for the dextran in comparison to LYCH (diffusion coefficient *D* ∝ 1/*R*_h_) and occlusion of flow in the apoplast by the dextran itself [50–52]. As was the case with LYCH, no detectable uptake of FITC-dextran occurred in mock deliveries (S2 Fig). Together, the experiments with LYCH and FITC-Dextran demonstrate that VACNFs promote direct delivery of probes into epidermal cells, apoplast, and in some cases palisade cells. Additionally, the mobility (or immobility) of delivered molecules is consistent with compartmental size-exclusion limits.

### VACNFs promote rapid delivery of proteins into plant tissue

**Fig 5:**
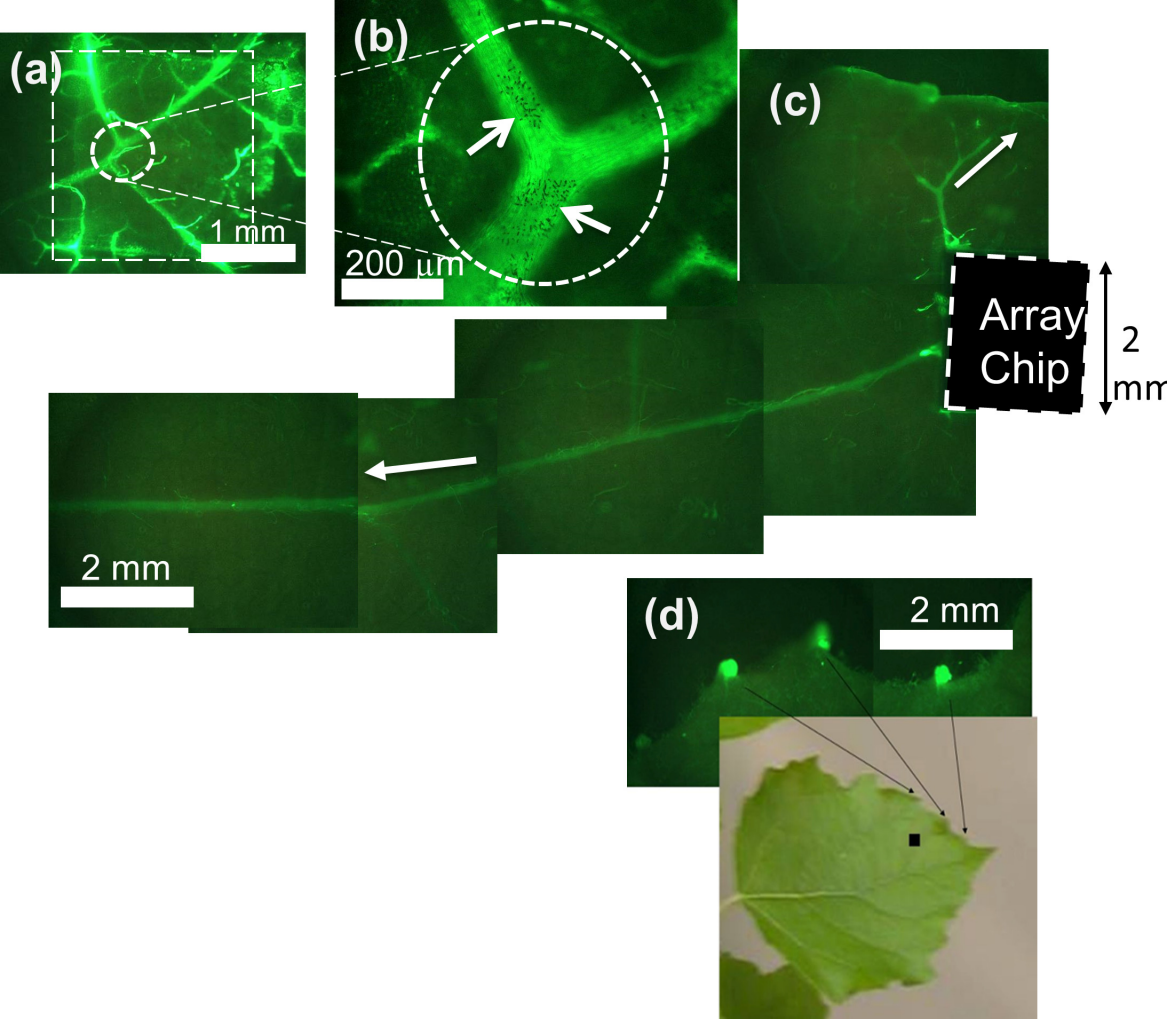
Carbon nanofiber arrays deliver fluorescent OG-CmPP16-1 into a *Populus* leaf. (a) Epifluorescence micrograph of the impaled region. The outline of the VACNF chip (ca. 2 × 2 mm) is indicated by the large dashed box. (b) Close-up view of a leaf vein junction showing carbon nanofiber spikes resident in the leaf. Arrows indicate clusters of fibers, in which individual fibers are visible as small black dots. (c) Composite epifluorescence image showing bidirectional movement of the labeled protein from the site of impalement with the VACNF array. The images were recorded 5–10 min after introduction of OG-CmPP16-1. Results shown are representative of 5 separate experiments (one plant per experiment). (d) Accumulation of fluorescence at the hydathodes was observed in 2 of 5 experiments 30 min after VACNF delivery. The black square indicates the location of the VACNF chip.

**Fig 6:**
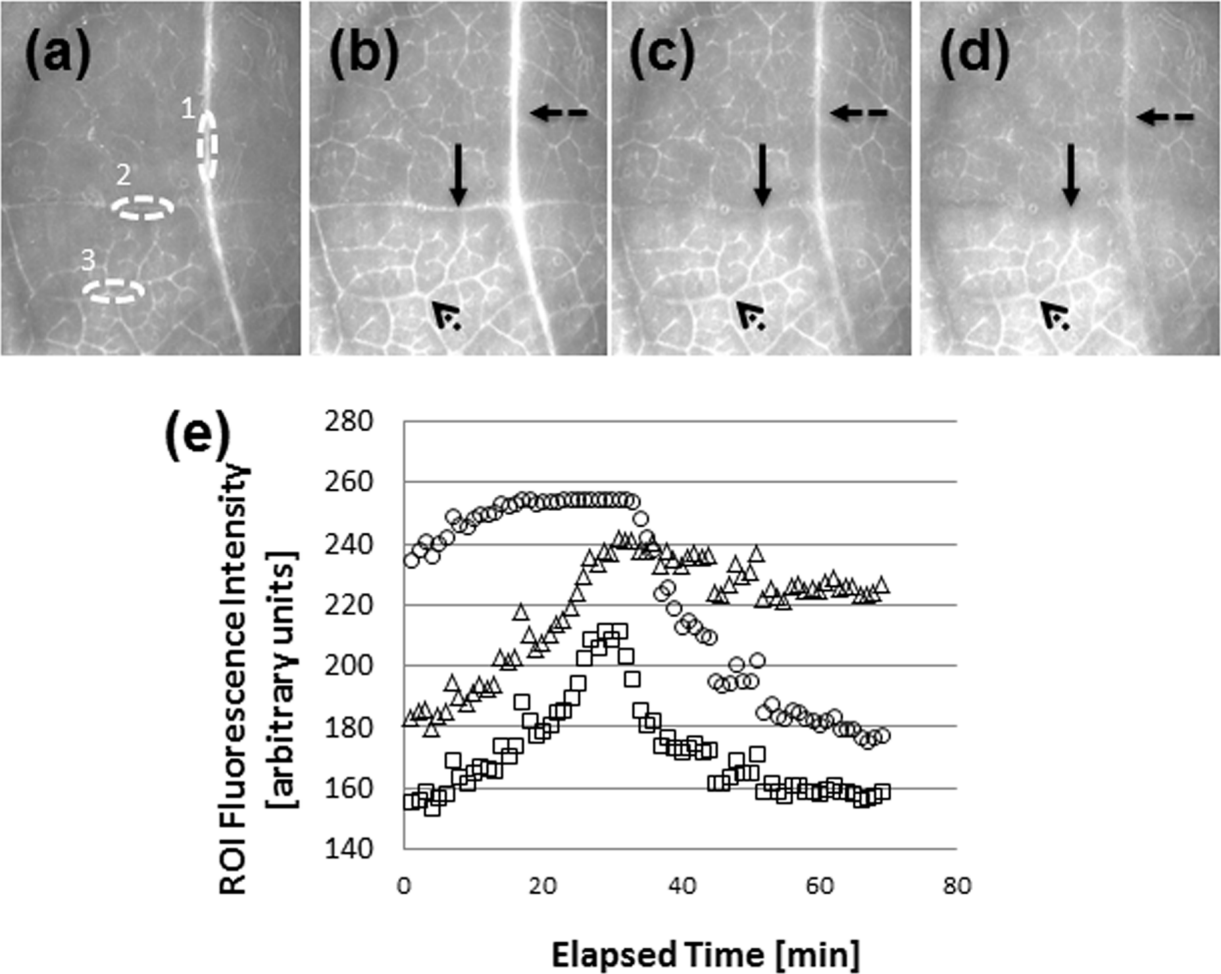
OG-CmPP16-1 moves through local *Populus* leaf vasculature following carbon nanofiber delivery. (a–d) Epifluorescence micrographs and (e) measured intensity at selected regions of interest (ROI). Fluorescence intensity was monitored at a main vein, a secondary vein, and a tertiary vein following introduction of the protein via a VACNF array. Veins are marked with numbers (1, 2, and 3, for the main, secondary and tertiary veins respectively) in (a) and with different styles of arrows in (b)–(d). Fluorescence micrographs were recorded at (a) 0 min, (b) 32 min, (c) 35 min, and (d) 42 min after VACNF delivery. In (e), fluorescence intensity is plotted for the main vein (circles), secondary vein (squares) and tertiary vein (triangles). The VACNF chip is located ~1 cm below and to the right of the imaged area. No correction for photobleaching was applied. Results shown are representative of 5 separate experiments (one plant per experiment).

Since molecules of widely varying size (0.5–500 kDa) could be delivered to leaf tissue and retained within cells in a size-dependent manner, we tested whether small proteins could move from the delivery site to distal regions of the leaf and whole plant. The cucurbit phloem-mobile protein CmPP16-1 (16 kDa) was used as a model for this study. A sample of the protein labeled with Oregon Green 488 (OG-CmPP16-1) was delivered into a *Populus* leaf still attached to the plant and observed by epifluorescence microscopy (Fig 5). No delivery of OG-CmPP16-1 into peripheral tissue was detected in control experiments in which a protein droplet was simply dispensed onto the leaf surface (data not shown). In contrast, OG-CmPP16-1 was rapidly delivered through the adaxial surface and into the vasculature after fiber impalement (Fig 5). Time-lapse imaging revealed that within minutes of the delivery, labeled protein had moved into major and minor veins around the delivery site (Fig 5A). Labeled protein always moved towards the main vein for transport into the plant proper (Fig 5C and 5D). In some cases (2 out of 5), we also observed movement towards the leaf edge, with accumulation at the hydathodes 20–40 mm distal from the point of introduction. While labeled protein could be observed throughout the vasculature of the treated leaf, none could be detected in distal leaves, and fluorescence could not be detected in the opaque parts of the plant (petiole and stem). Time-lapse imaging often revealed fluorescence loading of local vasculature beyond the perimeter of the delivery site that would progress for several tens of minutes before peaking and fading from the field of view of the microscope (Fig 6). Loss of fluorescence intensity likely reflects a combination of reduced delivery from the chip, transport out of the field of view and photobleaching.

### VACNFs enhance whole-plant delivery of ^125^I-CmPP16-1 into *Populus*

**Fig 7:**
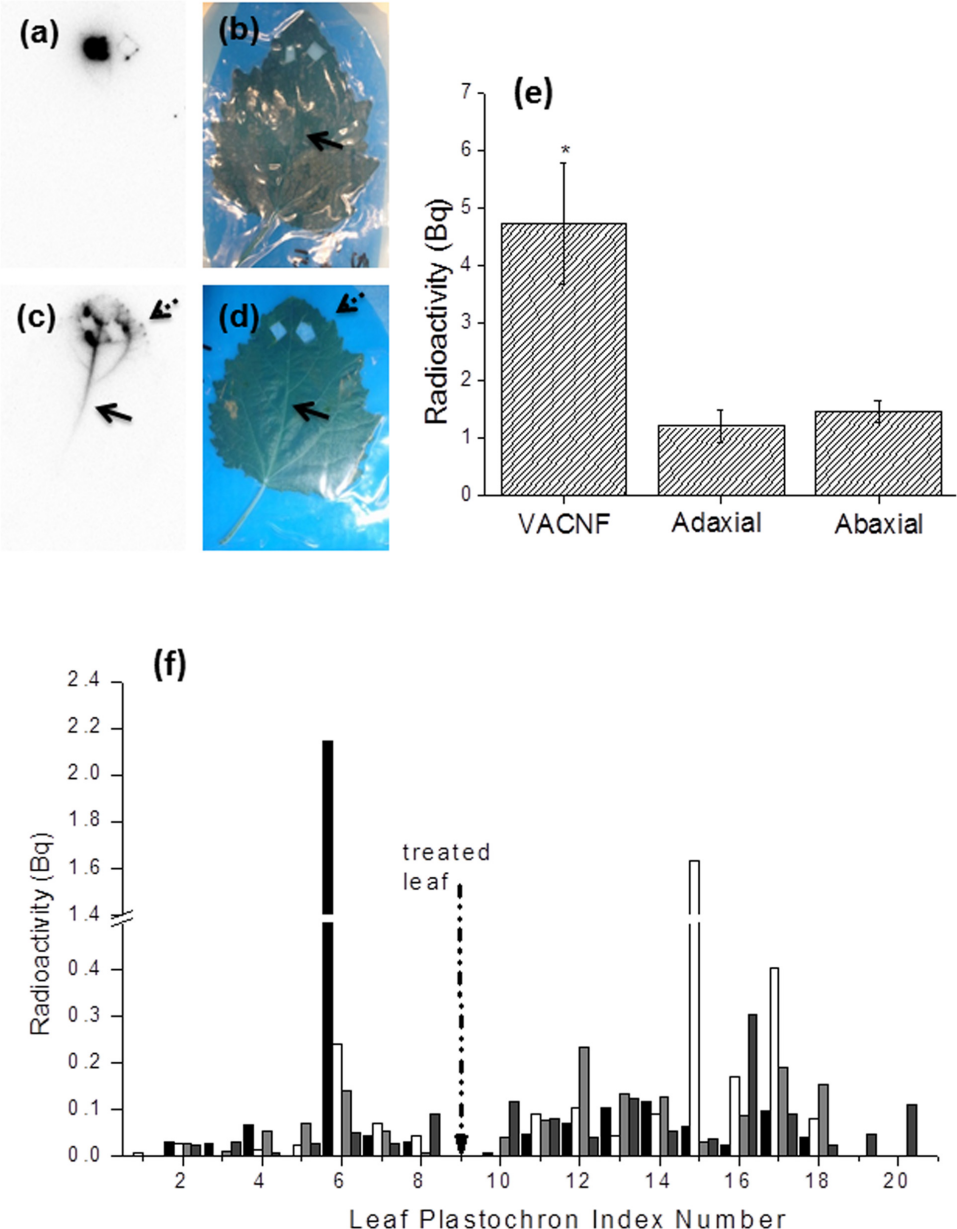
Carbon nanofiber arrays deliver ^125^I-CmPP16-1 into a *Populus* leaf and increase distribution to the whole plant. (a) Autoradiograph showing little or no movement from the site of application 5 min after passive delivery. (b) Photograph of the leaf in (a). (c) Autoradiograph showing movement of ^125^I-CmPP16-1 (2.3 μM, specific activity 3.4 × 10^5^ Bq/μg) into the vasculature of a leaf (LPI # 6) 5 min after VACNF delivery. The dashed arrow indicates accumulation of labeled protein at the hydathodes. The solid arrow shows movement down the main vein towards the petiole. (d) Photograph of the leaf in (c) with arrows indicating the mid-vein and leaf edge where hydathodes are located. The treated areas (near the top of the leaves) were excised so that the large amount of radioactivity associated with the chips and underlying leaf surface would not obscure surrounding areas in the autoradiographs. Excised areas are evident on autoradiographs (a and c) as diamond shapes that match the diamond cut-outs in the leaves. Autoradiographs in (a) and (c) were exposed for 21 h and are representative of two experiments with one plant per treatment. (e) Adaxial delivery via carbon nanofiber arrays increases uptake of ^125^I-CmPP16-1 by *Populus* plants as compared to passive droplet delivery on either the abaxial or adaxial leaf surfaces.^125^I-CmPP16-1 (3.3 μM, specific activity 3.2 × 10^4^ Bq/μg) was applied to a post-transitional leaf LPI #9 for 2 h using each delivery method. The plant was dissected, and accumulation of ^125^I in the leaves and stems (excluding the treated leaf) was measured. Data shown reflect the mean and SEM (error bars) of 5 separate experiments, each of which used two plants per VACNF treatment and one plant each per abaxial and adaxial passive treatment. Unpaired ANOVA followed by a Tukey multiple comparisons post-test showed that VACNF delivery was significantly greater than adaxial passive delivery at p<0.05 and is indicated by an “*” on the graph. When compared to abaxial passive delivery, the difference approaches significance with p = 0.067. (f) Leaf radioactivity counts from a representative experiment using two plants treated with the VACNF array on the adaxial surface and one plant each treated with passive droplets on the adaxial or abaxial surface. VACNF delivered ^125^I-CmPP16-1 moves both acropetally and basipetally from the point of delivery in *Populus* sapling leaves. Black bars, VACNF # 1; white bars, VACNF #2; light gray bars, adaxial droplet; and dark gray bars, abaxial droplet. The dashed arrow indicates the position of the treated leaf (LPI #9, radioactivity not measured).

We also examined the behavior of radiolabeled ^125^I-CmPP16-1, since the ɣ-emitting ^125^I can be imaged through optically opaque tissue and detected at very low levels. Leaves were treated with ^125^I-CmPP16-1 solutions at two sites on the leaf symmetrically flanking the mid-vein near the apex. Delivery was continued for 5 min, after which the leaves were removed from the plant, sealed in plastic, and autoradiographed. Results were consistent with those from the fluorescent probe. No movement of protein into the vasculature was observed after passive droplet delivery (Fig 7A). With nanofiber delivery, movement of the labeled protein into the mid-vein and secondary veins was apparent (Fig 7C). As with the fluorescent protein, the radiolabeled protein moved proximally, down the mid-vein toward the petiole, and sometimes distally, accumulating at hydathodes.

**Fig 8:**
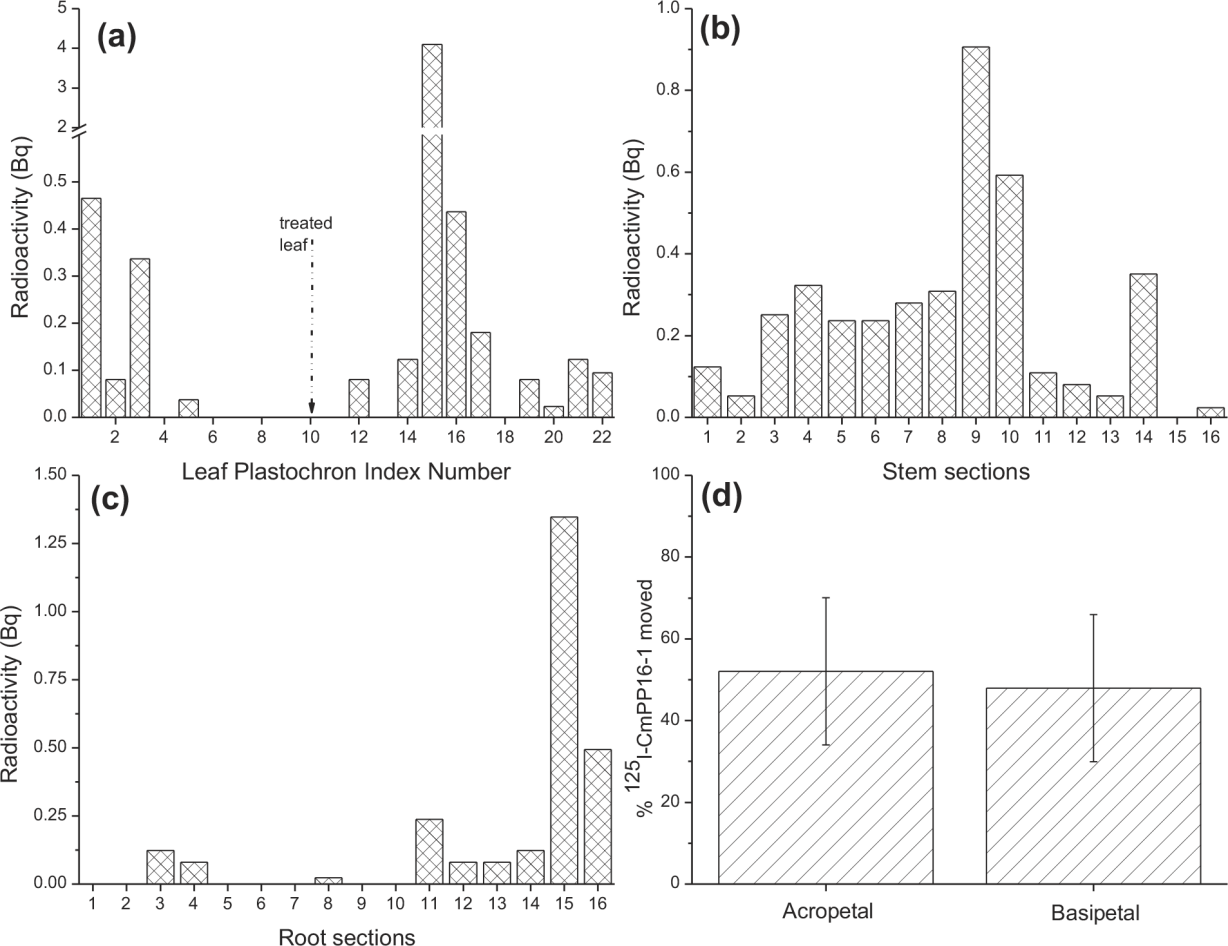
^125^I-CmPP16-1 delivered via carbon nanofiber arrays redistributes throughout *Populus* plants. Results shown reflect three replicate experiments (one plant per experiment). Plants were treated with 2 μL of 3.3 μM ^125^I-CmPP16-1 solution (specific activity 3.2 × 10^4^ Bq/μg) using VACNF arrays for 20 h, dissected and radiation-counted. Individual plants often show asymmetric distributions. (a)–(c) Show radiation counts from a single, representative experiment. (a) Leaves; the apical leaf is LPI #1, and the treated leaf is LPI #10 (marked by the dashed arrow and not counted). (b) Stems; stem sections were numbered from apex (1) to base (16). Stem section #9, with the highest level of radioactivity, includes the point of attachment of the treated leaf. (c) Roots; root sections were numbered from the top (1) to the tip (16). The label accumulates at the root tip (sections 14–16). (d) Mean accumulation of radioactivity in the plant relative to the site of VACNF delivery (error bars, SEM). On average, ^125^I-CmPP16-1 moves acropetally and basipetally in roughly equal amounts.

Success with delivery into the leaf vasculature led us to examine long-range protein movement of a radiolabeled probe throughout the entire plant. Initially, we treated plants for 2 h with ^125^I-CmPP16-1 and measured radioactivity in all parts of the plant outside the treated leaf. For this purpose, each leaf was removed and counted separately, stems were sectioned, and in some instances roots were also sectioned after washing to remove soil. Control experiments were performed with passive droplet delivery to either side of the leaf, as well as mock fiber delivery using smooth, fiber-free silicon chips. As shown in Fig 7E, the amount of radioactivity entering the plant from the treated leaf was 3 times larger with VACNF-mediated delivery than for the controls. This value, which includes leaves and stems, represents the average of 5 replicate experiments and is significant at the level of p < 0.05 for the adaxial control, which is the closest point of comparison, and p = 0.067 for the abaxial control. In replicate experiments with VACNF delivery, both basipetal and acropetal movement were observed (Fig 7F), but the fractions moving each direction varied,

Relatively small amounts of the radioactive probe moved outside of the treated leaf with the 2 h delivery, ~0.1% of the ^125^I-CmPP16-1 added. Therefore, we performed three additional experiments in which delivery via VACNF array was maintained for 20 h (Fig 8). Increasing the duration of the experiment did not significantly affect uptake of the radiolabeled protein, but there was more time for ^125^I-CmPP16-1 to redistribute within the plants. In these experiments, radioactivity moved throughout the plant, from root tip to apex (Fig 8). Radiolabel was detected in all parts of the stem, with greater amounts in stem sections near the treated leaf (Fig 8B). Radiolabel was also detected in most leaves, with the least being found near the treated leaf, possibly because the nearest neighbor leaves are non-orthostichous (Fig 8A) [53, 54]. In the roots, the protein moved all the way out to the tips (Fig 8C). These experiments confirm the bidirectional mobility of proteins introduced via VACNF. While ^125^I-CmPP16-1 consistently moved throughout the plant, the relative amounts moving basipetally and acropetally were variable (Fig 8D).

### CmPP-16-1 and GFP traffic differently in *Populus* after VACNF co-delivery

**Fig 9:**
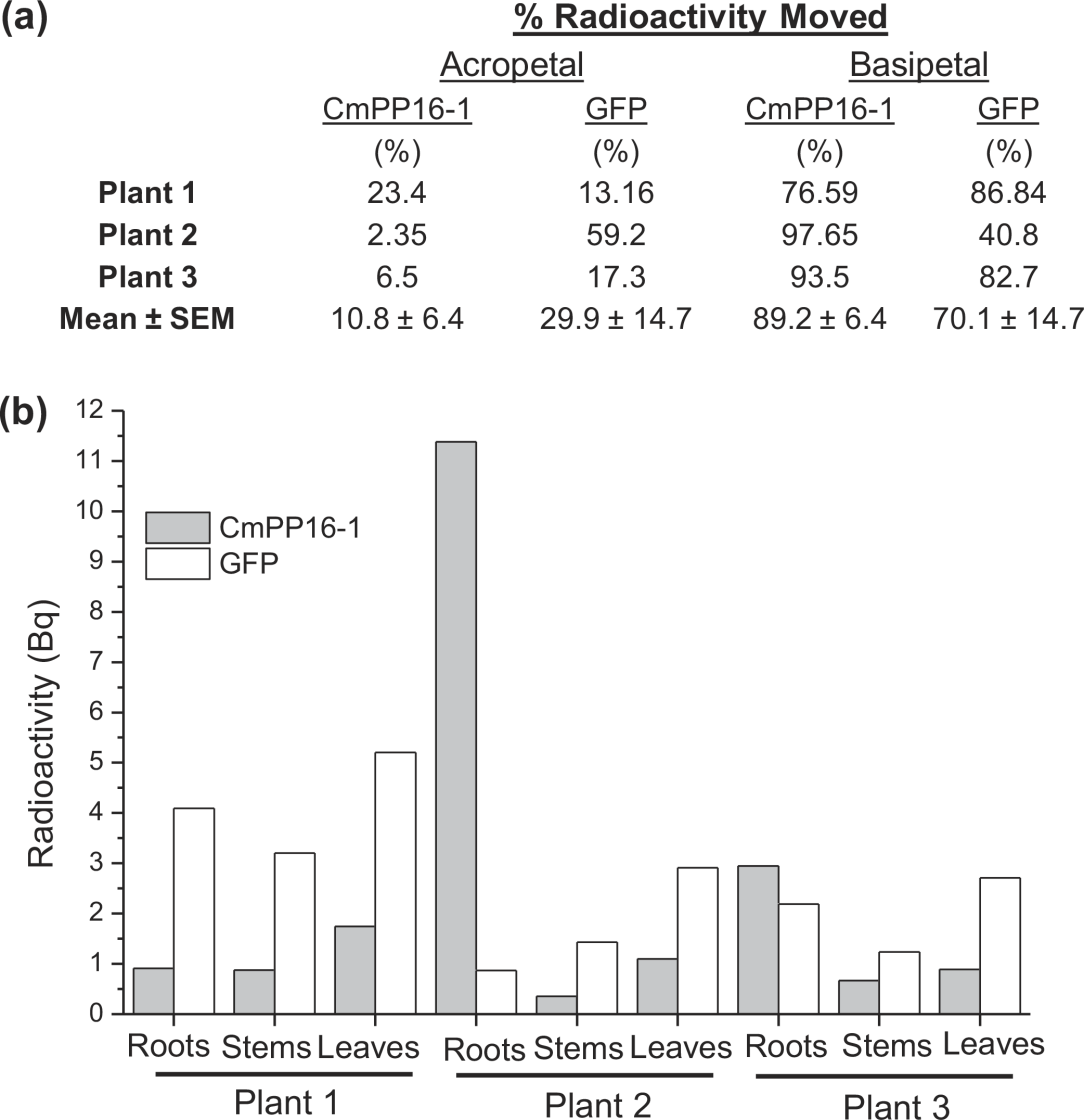
Plant and non-plant proteins distribute throughout the whole *Populus* sapling after VACNF delivery. In these experiments, individual plants were treated with 2 μL of a mixture of ^125^I-CmPP16-1 (4.5 x 10^3^ Bq/μL) and ^131^I-GFP (3.9 x 10^3^ Bq/μL), using VACNF arrays for 1 h, then dissected and radiation-counted. (a) Comparison of the % radioactivity (excluding the treated leaves) for ^125^I-CmPP16-1 and ^131^I-GFP that moved acropetally and basipetally within individual plants along with the mean and SEM for the 3 plants is tabulated. (b) Analysis of individual plants shows variability in the relative distribution of ^131^I-GFP and ^125^I-CmPP16-1 within the same plant.

To test the generality of VACNFs for delivery of proteins, and to determine whether the observed mobility of CmPP16-1 was unique, we co-delivered it with the small non-plant protein GFP. For these experiments, two radioiodine isotopes, ^125^I and ^131^I, were used. These isotopes afford probes that are chemically identical but that can be readily distinguished by differences in their half-lives and the decay energies of their γ-rays. Thus, ^125^I-CmPP16-1 was mixed with ^131^I-GFP and delivered to the leaf epidermis using VACNFs as above. Within five minutes, both ^125^I-CmPP16-1 and ^131^I-GFP entered the leaf vasculature (Fig 5C). After 1 h, both proteins could be detected throughout dissected plants (Fig 9A and 9B). No significant difference was observed in the average amounts of the two proteins that moved acropetally or basipetally throughout the plants (Fig 9A). We did, however, observe differences in the relative distribution of these proteins within individual plants (Fig 9B), indicating differences in the mechanisms of translocation, retention or both.

### Radiolabeled proteins are stable *in planta*

**Fig 10:**
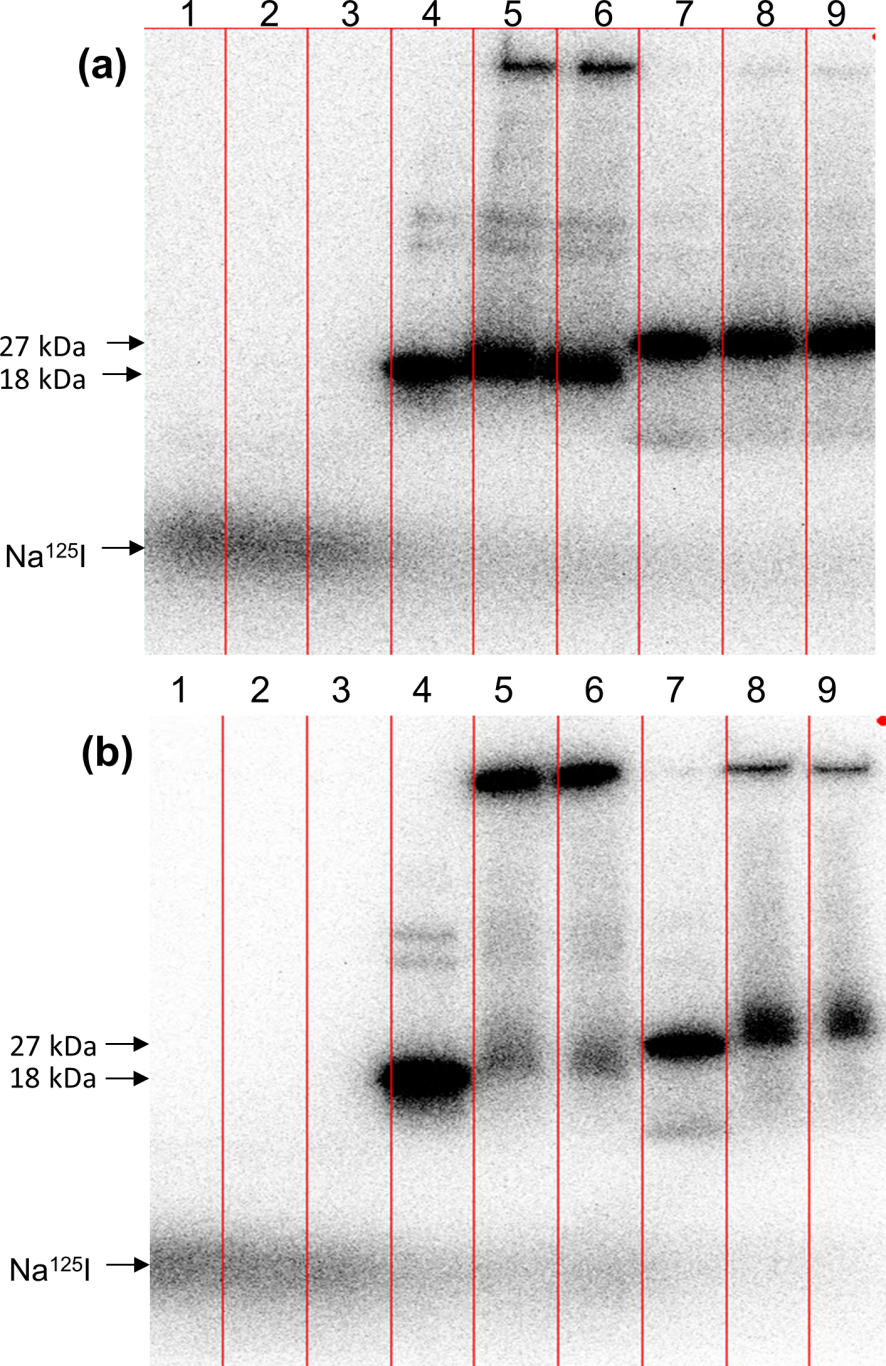
^125^I-CmPP16-1 and ^125^I-GFP retain their labels after exposure to leaf extract. ^125^I-CmPP16-1 (~6 x 10^3^ Bq), ^125^I-GFP (~2.9 x 10^3^ Bq) and Na^125^I (~2.1 x 10^3^ Bq) were incubated with whole leaf extract for (a) 2 h or (b) 20 h at room temperature prior to analysis by SDS–PAGE followed by autoradiography. Lane 1, pure Na^125^I; lanes 2 and 3, Na^125^I + whole leaf extract; lane 4, pure ^125^I-CmPP16-1; lanes 5–6, ^125^I-CmPP16-1 + whole plant extract; lane 7, ^125^I-GFP; lanes 8–9, ^125^I-GFP + whole plant extract. Autoradiographs were exposed for 20–25 minutes, and results shown are from single experiments.

An important consideration with labeled probes is assuring that the label (e.g., radiolabel or fluorophore) remains attached to the probe, and that the probe itself is not degraded. As the amount of probe introduced via VACNF-promoted delivery was too small to allow recovery of the protein for analysis, we performed *in vitro* stability studies using radiolabels, which can be detected with high sensitivity. ^125^I-CmPP16-1, ^125^I-GFP and Na^125^I were incubated with *Populus* whole leaf lysates for 2 h or 24 h and analyzed by SDS-PAGE followed by autoradiography to determine whether the radiolabeled-proteins remained intact. These experiments showed that after 2 h, most of the ^125^I-CmPP16-1 and ^125^I-GFP ran at the correct molecular weight, indicating that the label remained attached to the protein, and that the protein itself remained intact (Fig 10A). However, some of the label was associated with immobile material, presumably insoluble plant matter to which the proteins adhered. Over time more of each protein became associated with immobile plant material (Fig 10B). As an additional test, ^125^I-CmPP16-1 was introduced into *Populus* leaves by petiole feeding for 2 h or 20 h. Small leaf discs (~6 mm diameter) were excised from the treated leaves, homogenized in 2% SDS in PBS and analyzed by SDS–PAGE followed by autoradiography. The ^125^I-CmPP16-1 extracted from the leaves had not lost its label and appeared to be intact, although it was increasingly associated with large molecular weight immobile material after up to 20 h *in planta* (S3 Fig). While these experiments do not rule out the possibility of metabolic transformations, they do show that radioiodine is not readily cleaved from labeled proteins and that ^125^I-labeled CmPP16-1 and GFP remain stable in leaves for extended periods.

## Discussion

Understanding the role of biomolecules both locally and systemically within plants is of vital interest to yield advances in such areas as crop development, biomass production and plant pathogen interactions. To study these phenomena, it is helpful to introduced labeled molecules (probes) into particular parts of plants. Often, the greatest obstacle in this regard is circumventing the plant's natural barriers to deliver probes into the desired part of the plant with minimal perturbation. Genetic methods are useful for any probe that can be expressed (e.g., GFP-tagged proteins), while physical methods are more versatile but can wound or stress plants, potentially interfering with the effect under study. Here, we have demonstrated that carbon nanofiber arrays can be used to deliver molecules of varying size, including LYCH (0.5 kDa, hydrodynamic radius *R*_h_ = 0.68 nm [30]), small proteins (~15–30 kDa, *R*_h_ ≈ 2–2.5 nm) and fluorescent dextran (500 kDa) directly into epidermal cells in *Populus* leaves. Delivery was also observed to some palisade cells, via presumed transient overpenetration of the epidermis, and to apoplast, likely via intercelullar fiber penetration but possibly also due to leakage or diffusion out of cells. The large 500 kDa FITC-dextran was retained in epidermal and palisade cells, with no lateral migration observed between cells or in apoplast. LYCH was more mobile but was restricted predominantly to impaled epidermal cells, underlying palisade cells and the surrounding apoplast. The dye did not apparently migrate from impaled cells into adjacent unimpaled ones. It is unknown whether this symplastic isolation is the natural condition, or is the result of temporary loss of turgor pressure in the impaled cells [33]. The integrity of the cells must be compromised transiently to allow entry of exogenous molecules. However, with VACNF arrays, cells do not collapse or show any other overt damage, and any wound response is below detection limits as determined by DAB staining for H_2_O_2_ production. We conclude that carbon nanofibers offer a minimally disruptive method for delivery of biomolecules to discrete surface areas on leaves.

Delivery occurs via multiple small entry points into the leaf, created by the nanofiber arrays, each of which likely delivers sub-nanoliter quantities of labeled protein to a single cell or a single point in the intercellular space. The mechanism of delivery has not been determined, but several non-exclusive possibilities can be considered. In one scenario, the plasma membrane remains tightly sealed around the fibers, and molecules are delivered during impalement and subsequently by desorption from the surface of the intracellular part of the fibers. In a second scenario, sealing is imperfect around the fibers for a significant time period, leaving channels through which molecules can diffuse into cells. In a third scenario, impalement causes an open wound through which molecules enter and from which the cell might or might not recover. Our results are best explained by the first two scenarios. Experiments with LYCH and FITC dextran suggest that initial uptake from around the spikes is rapid (< 5min), whereas other experiments show that its duration is finite. In fluorescence-tracer experiments with OG-CmPP16, delivery to regions around the impalement site peaked at around 30 minutes. In the radio-tracer experiments, only a small fraction (~0.1%) of the total activity exited the treated leaf and moved to distal tissue, and not much more moved after 20 h than after 2 h. Taken together, these observations indicate that permeablization is transient, though we cannot determine whether delivery in the minutes after impalement results from leakage around the fibers or desorption from them. Previous studies have shown that plant cells reseal and reestablish membrane potential within a few minutes after microinjection [55], and a similar time period for resealing around the nanofibers is plausible.

Small proteins, such as GFP and the cucurbit phloem protein CmPP-16-1, introduced using VACNFs traveled bidirectionally, both within the treated leaf and within the stem of the plant, reflecting uptake into both xylem and phloem. The xylem-delivered fraction likely loads from the apoplast, whereas the proteins within the phloem most likely load through the symplast, particularly given that our plant model was *Populus*, a passive loader with many symplastic connections between the cells [56, 57]. The use of VACNFs increased delivery of CmPP16-1 to *Populus* relative to passive uptake. Both CmPP16-1 and GFP delivered via VANCF moved to similar extents within the plant but did not co-migrate completely. It is unknown whether movement of these proteins largely followed bulk flow or was directed in some way. CmPP16-1 is believed to move in complex with other proteins and potentially RNA in phloem [58, 59]. Whether analogous partners bind CmPP16-1 or influence its transport in *Populus* remains to be determined. GFP is a non-plant protein and hence presumably lacks specific partners, and its migration is more likely to follow bulk flow.

An ideal delivery method would allow controlled introduction of the desired molecule at any point on the plant without significant perturbation of the plant physiology. Lack of such an ideal method drives the push for new approaches that offer improvements in one or more respects, such as generality, ease of use, selectivity, scalability, or invasiveness. As illustrated by both our work and the recent work of Etxeberria et al. [25], transfer of new technologies from one application to another can foster progress on long-standing problems. Thus, both laser marking techniques and carbon nanofibers, though not originally developed for permeablizing plant tissue, can be advantageously employed for the purpose. Our study demonstrated specifically that arrays of carbon nanofibers 20–25 μm long can penetrate the cuticle, cell wall and plasma membrane of leaf epidermal cells in *Populus* to introduce molecules of widely varying size directly inside the cells and can on occasion pierce multiple cell walls to deliver to the underlying palisade mesophyll cells. Key benefits to the VACNF approach include simplicity, flexibility in the site of introduction, and ability to form multiple penetrations in a small area for delivery of greater quantities of probe. In addition, carbon nanofibers are inert, and they deliver only the molecule of interest without compromising cell integrity or eliciting a wound response. VACNF arrays can be made in most university clean rooms, and turn-key systems for fiber growth via PECVD are now commercially available. Arrays can also be obtained through the user program at the Oak Ridge National Laboratory Center for Nanophase Materials Sciences (CNMS). Support from CNMS is awarded through a peer-reviewed proposal system and is provided at no cost to successful applicants who intend to publish their results [60]. The primary limitation of the technique is its lack of specificity with regard to site of delivery (i.e., intra- or inter-cellular), an issue also shared by other physical delivery methods that deliver to multiple cells simultaneously.

While this study has shown proof of concept for the use of VACNF arrays, the full potential of carbon nanofiber technology for microdelivery to plants is yet to be realized. The deterministic nature of fiber growth allows great freedom in both the spatial positioning of fibers and their height. Through optimization of pattern, pitch and height, along with impalement and treatment protocols, substantial improvement can likely be made over the results communicated herein. Minor adjustments to the nanofiber height, for example, could increase the extent of penetration into palisade cells, or ensure impalement of only the epidermal layer. Fibers can also be transferred to polymeric films to generate conformal arrays [61], and the capability to produce similarly sized hollow fibers (nanopipes) [62] may allow active coupling of the plant epidermis to microdevices, such that fluids can be both introduced and withdrawn for analysis, as by mass spectrometry. Extension to classes of molecules other than proteins should be readily achievable. Potential applications include the delivery of small molecules that promote or inhibit signaling, regulatory RNA (e.g. artificial microRNAs) or synthetic DNA constructs, as has been demonstrated with animal cells [28, 63]. Delivery of bacterial or viral vectors for research or biotechnological applications may also be feasible.

Even in its present early stage of development, the VACNF approach provides a unique method for delivering labeled, exogenous biomolecules directly into epidermal cells and indirectly into the plant vasculature. This capability provides a ready platform to examine local effects of biomolecule delivery to leaf tissue, for example, to regulate stomatal opening and closing. In conjunction with γ-emitting radiolabels such as ^125^I, the enhanced delivery promoted by VACNFs enables long-range mobility studies on trace analytes in larger, woody plants such as *Populus*. Thus, it can find immediate application in elucidating the role of long-distance biomolecule transport in plant development [64, 65], plant stress responses [66] and interaction with microbes [67].

## Materials and Methods

### Reagents and equipment

Except where otherwise stated, general chemicals and reagents were obtained from commercial suppliers. Molecular biology and cell lysis reagents were obtained from Novagen (EMD Millipore, Billerica, MA, USA). Na^125^I and Na ^131^I were obtained from Perkin Elmer (Waltham, MA, USA). An automated γ-ray scintillation counter consisting of a single 7.6-cm planar-type NaI(Tl) detector (Packard Quantum Cobra D5002) was used for assay of ^125^I and ^131^I. The detector discriminator window was set to bracket the 27–32 keV X-rays and 35 keV γ-ray of ^125^I and the 260–470 keV γ-rays of ^131^I. A spilldown correction factor of 14% was applied to account for the spillover of ^131^I into the ^125^I detection window. Samples were typically counted for 1 min. No corrections were made for the attenuation of the ^125^I X- and γ-rays by the samples.

### Plants and plant growth conditions

*Populus* plants, clonal ramets of a hybrid aspen (7171B4, female, *P*. *tremula × P*. *alba*), were established from stem cuttings potted in a mixture containing equal parts Fafard 4M (proprietary mixture), vermiculite and perlite in tall leach tubes. Plants were grown to a height of 30–45 cm in the greenhouse at 60–65% humidity and 23–25°C with a 16-h photoperiod; supplemental light was provided by metal halide bulbs at a photon flux density of 45 μmol m⁻^2^ s⁻^1^.

### Protein purification and labeling

CmPP16-1 cDNA was amplified by PCR using the following primers: forward primer, 5’-GGAATTCCATATGATGGGGATGGGAATG-3’, and reverse primer, 5’-GAAAGGGATCCTTAGTTTTCCCATGG-3’, and cloned into pET15b (Invitrogen). After sequence verification, the resulting plasmid was transformed into *Escherichia coli* BL21(DE3). Transformants were grown with shaking at 37°C in LB medium to mid-log phase (OD_600_ ≈ 0.6) and induced with 1 mM IPTG for 4 h. His-tagged CmPP16-1 was purified from lysed cells using Ni-NTA affinity beads (Sigma-Aldrich, St. Louis, MO, USA) according to the manufacturer’s instructions. The eluted protein was dialyzed against PBS over 24 h with 3 exchanges of buffer using Spectra/Por 7 dialysis membrane with a molecular weight cut-off (MWCO) of 7 kDa (Spectrum, Rancho Dominguez, CA, USA). Similarly, eGFP was cloned into pDEST17 (Invitrogen) and purified using Ni-NTA affinity beads as described [68].

For fluorescent labeling, purified CmPP16-1 was tagged using Oregon Green 488 5-carboxylic acid, *N*-hydroxysuccinimidyl ester (Invitrogen) according to the manufacturer’s instructions, affording OG-CmPP16-1. In a typical radioiodination, protein (50–55 μg) in PBS (56–70 μL) was labeled with either 1 or 10 μL of sodium radioiodide of specific activity 3.7 × 10^6^ Bq/μL and chloramine-T [69]. Labeled proteins were separated from unreacted iodine using 5-mL Ultrogel AcA 44 columns (Sigma-Aldrich) equilibrated with 5 mg/mL of BSA in PBS. The specific activity for experiments using CmPP16-1 alone was 3.2 × 10^4^ Bq/μg (1 μL of Na^125^I) and 3.5 × 10^5^ or 3.8 × 10^5^ Bq/μg (10 μL of Na^125^I). For the lower specific activity CmPP16-1 sample, a 400-μL aliquot of the labeled protein was concentrated to 150 μL using a Microcon centrifugal concentrator (EMD Millipore) with a molecular weight cutoff of 3 kDa, giving a final concentration of 3.3 μM.

For co-mobility experiments involving CmPP16-1 and GFP, specific activities were 6.25 × 10^5^ (^125^I-CmPP16-1), and 3.07 × 10^5^ (^131^I-GFP) Bq/μg from labeling with 10 μL of NaI. GFP was also labeled with Na^125^I at a specific activity of 2.9 × 10^5^ Bq/μg for labeled protein stability studies. A sample from each reaction was analyzed by SDS–PAGE followed by autoradiography (below) to confirm labeling.

### Autoradiography

Samples for autoradiography were sealed in plastic and exposed to either multi-sensitive or super-resolution phosphor screens (Perkin Elmer) for exposure times ranging from 20 min to ~5 days in photographic cassettes, at 4°C. Screens were scanned using a Cyclone Plus PhosphorImager (Perkin Elmer). Image capture and export was performed using Snagit^™^ software (TechSmith Corp., Okemos, MI, USA).

### Synthesis of vertically aligned carbon nanofiber arrays

VACNFs were synthesized on a solid substrate as previously described [70]. In brief, photolithography and electron-beam evaporation were used to pattern 50 nm thick × 500 nm diameter dots of nickel on a square grid with a lateral spacing (pitch) of either 10 or 20 μm across the surface of a silicon wafer. Nanofibers were grown at the nickel dots in a plasma-enhanced chemical vapor deposition (PECVD) chamber fed with an acetylene/ammonia mixture. After growth, the wafer was spin-coated with a protective layer of photoresist and diced into 2 × 2 mm square chips, each of which contained ~40,000 fibers (10-μm pitch) or ~ 10,000 fibers (20-μm pitch). Array quality and fiber dimensions were assessed by scanning electron microscopy (SEM) using a Hitachi S-4700 scanning electron microscope. Samples were affixed to the SEM stage with carbon tape and imaged at a 30° tilt with an acceleration potential of 10 kV. Prior to use, each chip was stripped of photoresist by washing for 300 s in *N*-methylpyrrolidone, followed by rinsing extensively in distilled water. The chips were further cleaned by a 15 s etch in a water-vapor inductively coupled plasma (Harrick Plasma Cleaner, Ithaca, NY, USA).

### Microdelivery with vertically aligned carbon nanofiber arrays

Typically, a 1-μL droplet containing the probe of interest was applied to the adaxial surface of the target leaf. A VACNF chip (2 × 2 mm) was placed on top of the droplet, and the fibers were pressed into the leaf tissue by tapping the chip gently with a pair of forceps, using a convenient surface (such as a microscope stage, microscope slide, or glass culture dish) to support the back of the leaf. Alternatively, the droplet was placed on the chip, and the chip was placed on the adaxial leaf surface. After the desired delivery period had elapsed, the chip was removed. For control experiments, the chip was applied with its smooth back to the leaf; in some cases, the tap on the control chip was omitted, in particular to prevent the chip from sliding and spreading radioactivity. Impaled leaves were examined by SEM. Prior to imaging, stage-mounted leaf tissue was placed in the sample exchange chamber and brought under vacuum. When leaves were substantially dehydrated and the pressure had dropped to ~2 Pa (~15 min), the specimen was transferred to the microscope main chamber. Imaging was conducted at a 30° tilt and an acceleration potential of 2 kV.

### Examination of epidermal cell permeability and wounding following VACNF treatment

The membrane-impermeant probes LYCH (Sigma-Aldrich) at 1 mM and 500-kDa–FITC-dextran (Invitrogen, ThermoFisher, Waltham, MA, USA) at 0.1 mg/mL were delivered to the adaxial surface of *Populus* leaves. After 5 min of uptake, the treated leaf was removed from the plant, and the chip area, along with ~2 mm of surrounding tissue, was excised. The chip was removed, and the leaf section was washed with PBS to remove surface fluorescence. The leaf segment was placed on a microscope slide, sealed under a coverslip with a mixture of petroleum jelly, lanolin and paraffin in a 1:1:1 ratio and immediately observed by confocal laser scanning microscopy using a Zeiss LSM 710 microscope.

To assess wounding, potentially damaging treatments were conducted on single *Populus* leaves attached to the plant. Leaves were (a) cut through with a cork borer, (b) dry-spiked using VACNFs or (c) abraded with 320-grit carborundum powder applied as a thin layer to a droplet of water on the leaf surface and gently brushed using a non-abrasive foam-tipped applicator (Fisher Scientific, Pittsburgh, PA, USA). H_2_O_2_ production, was assessed by staining with 3,3'-diaminobenzadine (DAB) [71, 72] as follows. After treatment, leaves were immediately severed at the base of the stem with a scalpel, and their petioles were placed in a 0.5 mg/mL solution of DAB for 2 h, after which the leaves were boiled in 95% ethanol for 10 min. Staining was assessed visually using bright-field microscopy with the Diaphot 300 microscope.

Mechanical damage was assessed by DIC microscopy. Transverse views were obtained from leaf tissue that had been fixed overnight in 10% neutral buffered formalin and dehydrated in 80% ethanol. Fixed tissue was embedded in paraffin, cut into 3–5 μm thick transverse sections and stained with toluidine blue by Ridge Microtome service (Knoxville, TN). Images were obtained with the LSM 710 laser scanning microscope operating in DIC mode. Longitudinal views were obtained from leaf tissue that had been fixed overnight in ethanol:acetic acid (9:1), serially dehydrated into 35% ethanol and cleared overnight in chloral hydrate solution (chloral hydrate: H_2_O: glycerol 8:1:1). Images were obtained using a Nikon Eclipse 80i microscope with DIC optics and a Nikon DXM-1200c 12-megapixel color camera.

### Local and long-distance movement of proteins in *Populus*

#### Local movement of OG-CmPP16-1 and 125I-CmPP16-1

OG-CmPP16-1 was diluted in 10 mM 2-(*N*-morpholino)ethanesulfonic acid (MES) buffer, pH 5.7, containing 10% sucrose and 1 mM EDTA to provide a final protein concentration of 0.25 μg/μL (13 μM). After impalement with VACNFs, time-lapse epifluorescence image sequences were recorded on the Diaphot 300.

For experiments with radiolabeled proteins, delivery was allowed to progress for 5 min. The leaves were then removed from the plant and sealed in thin (~50 μm), adhesive plastic film (Pinnacle Cover-All, Total Care, Orange, CA, USA). With a scalpel, the chip and ~1 mm of surrounding leaf tissue were excised; this step was necessary to prevent the large amount of radioactivity associated with the chip from obscuring the autoradiographs. The leaf was then resealed and secondarily contained in a plastic bag for autoradiography.

#### Long-distance movement of 125I-CmPP16-1 and co-mobility of 125I-CmPP16-1 and 131I-GFP

For each experiment, two plants were treated as described above with ^125^I-CmPP16-1 plus VACNF chips on the adaxial leaf surface, and two served as passive delivery controls, one each for the adaxial and abaxial surfaces of the leaf. On the adaxial surface, a fiber-free control chip was used, while on the abaxial surface, no chip was used. On each plant, a pair of droplets of ^125^I-CmPP16-1 solution (3.3 μM) were placed on the appropriate surface near the tip of fully expanded post-transitional leaves [54], followed by VACNF or control chips. After treatment, the plants were maintained at room temperature in the radiological fume hood under a fluorescent light for 2 h. All leaves were removed, starting with the treated leaf, and the stem was cut into sections approximating the internode distances on the plant. All samples were sealed in 12 × 75 mm plastic tubes, and the amount of ^125^I was measured. Results are expressed as the radioactivity measured within the plant leaves and stem after 2 h, excluding the treated leaf. In other experiments, post-transitional leaves on *Populus* plants were treated using VACNF delivery with ^125^I-CmPP16-1 and left for 20 h before dissection and radioactivity counting. Leaves were cut off and stems were sectioned as before, while roots were washed with water to remove soil and cut into sections 2.5–4.0 cm long.

For co-mobility studies, ^125^I-CmPP16-1 and ^131^I-GFP were pre-mixed to 0.38 μM and 0.47 μM respectively, before application. After VACNF-treatment, plants were maintained in the radiological hood for 1 h at room temperature prior to dissection and radioactivity counting.

### Analysis of radiolabeled protein stability *in planta*

To determine whether plant enzymes cleave iodine from radioiodinated proteins, 10 μL of crude leaf homogenate was incubated with 1 μL of ^125^I-CmPP16-1, ^125^I-GFP or Na^125^I for 2–24 h at room temperature. Briefly, ~0.25 g of leaf tissue was frozen in liquid nitrogen, ground with a mortar and pestle, resuspended in 1 mL of 0.2% IGEPAL-630 in PBS and centrifuged for 1 min at 4000 × g to pellet debris. Additionally, petioles of freshly-cut *Populus* leaves were immersed in ^125^I-CmPP16-1 for 2 h or 20 h petiole feeding. Discs (6 mm diameter) were removed from each leaf and extracted into 50–75 μL of 2% SDS in PBS by homogenizing for 30–40 s in a microcentrifuge tube using a Kontes polypropylene Pellet Pestle attached to a Dremel rotary tool, followed by heating at 95°C for 10 minutes. Samples were analyzed by SDS-PAGE followed by autoradiography.

## Supporting Information

S1 Fig LYCH does not penetrate the *Populus* leaf surface without VACNF assistance. LYCH dye (1 mM) was applied as a one microliter droplet to the adaxialsurface of a *Populus* leaf, a silicon chip without nanofibers was placed on top, without pressing, and left in place for 5 min. The treated leaf was then removed from the plant, the chip area was excised using a scalpel and the chip was removed. The leaf section was gently washed to remove surface LYCH dye and imaged by confocal microscopy. (a) Green channel, showing that epidermal cells evidenced no green (LYCH) fluorescence (b) Red channel, showing autofluorescence from chloroplasts (c) Transmitted light channel, showing the structure of the tissue. (d) Merged image of (a), (b) and (c), showing that unimpaled cells do not take up LYCH.
(TIF)

S2 Fig FITC-dextran does not penetrate the *Populus* leaf surface without VACNF assistance. FITC-dextran (500 kDa, 0.1 mg/mL) was applied to the adaxial surface of a *Populus* leaf, a silicon chip without nanofibers was placed on top, without pressing, and left in place for 5 min. The treated leaf was then removed from the plant, the chip area was excised using a scalpel and the chip was removed. The leaf section was gently washed to remove surface FITC-dextran and imaged by confocal microscopy (a) Green channel, showing that epidermal cells evidenced no green (FITC) fluorescence (b) Red channel, showing autofluorescence from a few chloroplasts (c) Transmitted light channel, showing the structure of the tissue. (d) Merged image of (a), (b) and (c), showing that unimpaled cells do not take up FITC-dextran.
(TIF)

S3 Fig ^125^I-CmPP16-1 is stable *in planta*. (a) And (c) show autoradiographs of protein recovered from *Populus* leaves after 2 h or 20 h, respectively, and separated by SDS–PAGE. (b) and (d) Show autoradiographs of leaves analyzed in (a) and (c), respectively. (a) ^125^I-CmPP16-1 (1.2 μM, specific activity 3.5 × 105 Bq/μg) was stem-fed into a *Populus* leaf (LPI #8) for 2 h, extracted from two discs (i and ii) taken from the leaf, separated by SDS-PAGE and autoradiographed for 2 h. Lane 1, pure ^125^I-CmPP16-1; lane 2, mixture of ^125^I-CmPP16-1 and pure Na^125^I; lane 3, extract from leaf disc (i); lane 4, extract from leaf disc (ii); and lane 5, Na^125^I. The upper and lower arrows indicate the positions of ^125^I-CmPP16-1 and Na^125^I, respectively on the gel. All lanes are from the same exposure of one continuous gel. The black bar between lanes 1 and 2 represents intervening lanes omitted here for clarity (the complete gel is provided as S4 Fig). (b) Autoradiograph (21 h exposure) of the leaf analyzed in (a). The arrows indicate the location of the leaf discs that were removed for extraction. Results shown are representative of two experiments. (c) ^125^I-CmPP16-1 (12 nM, specific activity 3.5 × 105 Bq/μg) was stem-fed into a *Populus* leaf (LPI #9) for 20 h, extracted from a disc taken from the leaf, separated by SDS-PAGE and autoradiographed for 20 h. Lane 1, pure ^125^I-CmPP16-1; lanes 2–5 contain extracts from discs cut from four different stem-fed leaves. The autoradiograph was exposed for 3.5 days. (d) Autoradiograph of a representative *Populus* leaf (LPI #9) after being stem-fed with 12 nM ^125^I-CmPP16-1 (specific activity 3.5 × 105 Bq/μg) for 20 h. This leaf corresponds to lane #4 on the gel. The autoradiograph was exposed for 3.5 days, and the arrow indicates the location of the removed leaf disc.
(EPS)

S4 Fig Complete autoradiograph used in S3 Fig (A). (TIF)

S5 Fig Complete autoradiograph used in S3 Fig (C). (TIF)

S6 Fig Complete autoradiograph used in Fig 10 (A). (TIF)

S7 Fig Complete autoradiograph used in Fig 10 (B). (TIF)
