# Supplementary figures and images for: Carbon Nanofiber Arrays: A Novel Tool for Microdelivery of Biomolecules to Plants
Source: PLoS One. 2016 Apr 27;11(4):e0153621. doi: 10.1371/journal.pone.0153621 (PMC4847769; doi:10.1371/journal.pone.0153621)

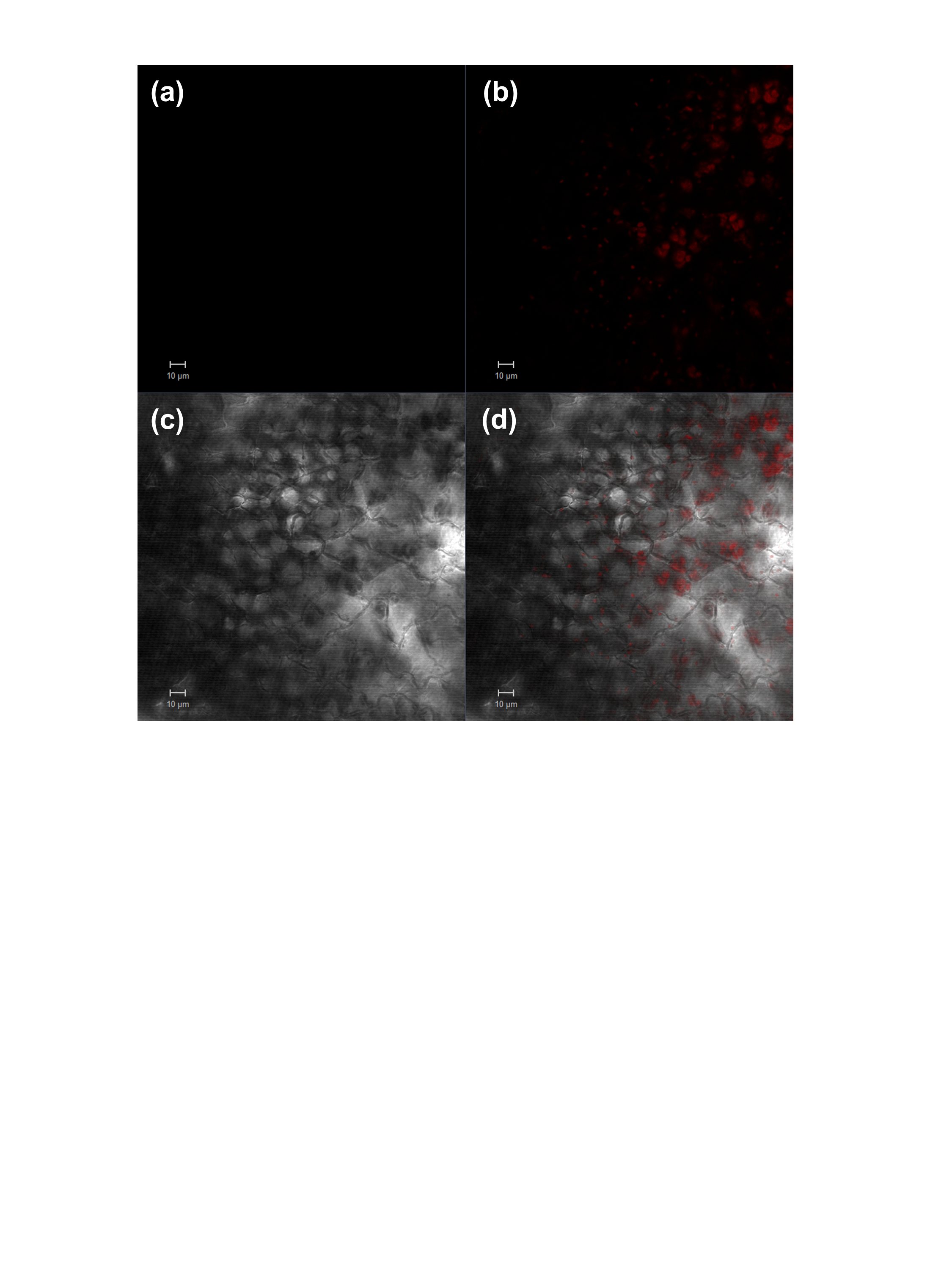

Supplement: S1 Fig — LYCH dye (1 mM) was applied as a one microliter droplet to the adaxialsurface of a Populus leaf, a silicon chip without nanofibers was placed on top, without pressing, and left in place for 5 min. The treated leaf was then removed from the plant, the chip area was excised using a scalpel and the chip was removed. The leaf section was gently washed to remove surface LYCH dye and imaged by confocal microscopy. (a) Green channel, showing that epidermal cells evidenced no green (LYCH) fluorescence (b) Red channel, showing autofluorescence from chloroplasts (c) Transmitted light channel, showing the structure of the tissue. (d) Merged image of (a), (b) and (c), showing that unimpaled cells do not take up LYCH. (TIF) [file pone.0153621.s001.tif]

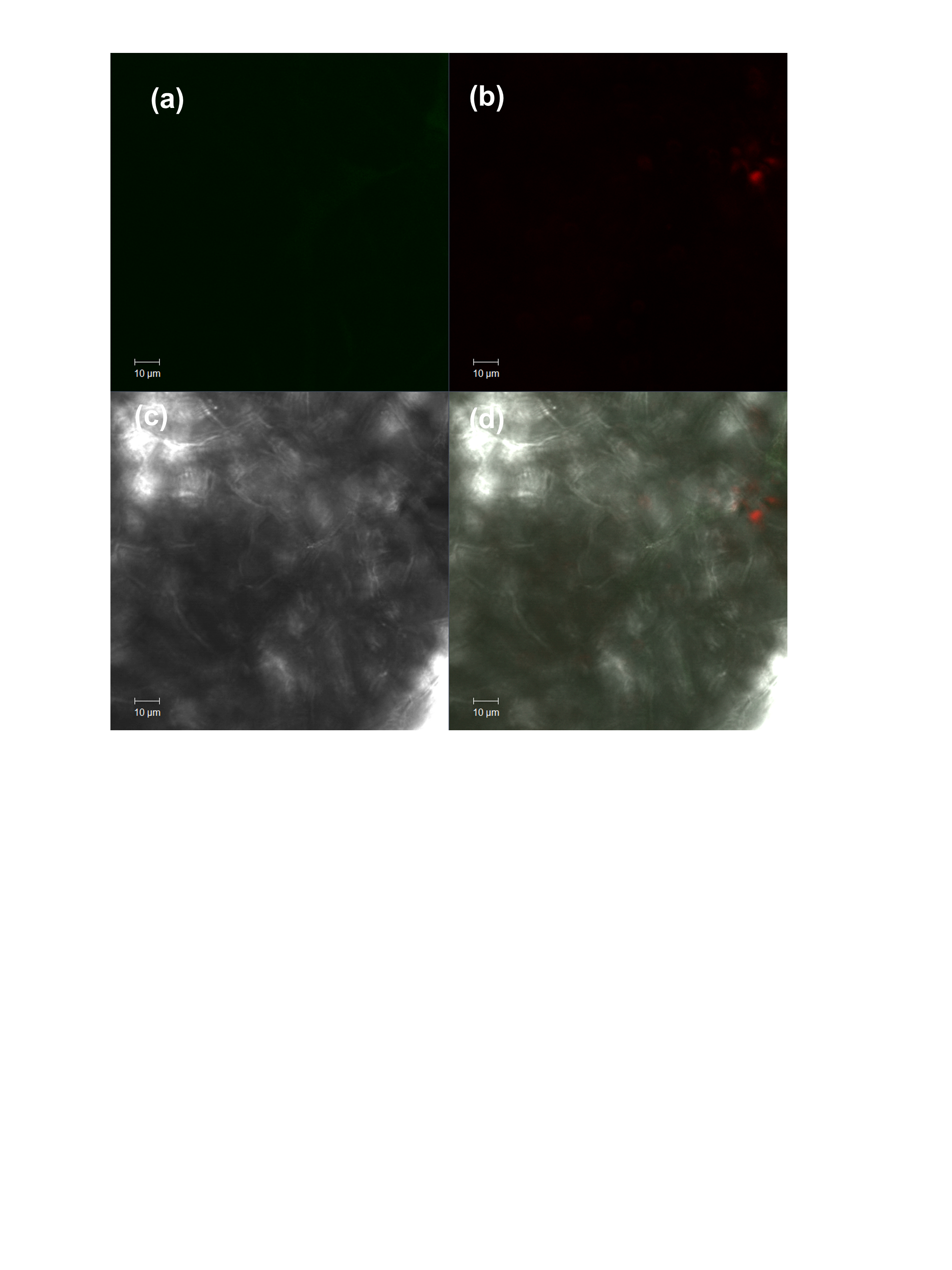

Supplement: S2 Fig — FITC-dextran (500 kDa, 0.1 mg/mL) was applied to the adaxial surface of a Populus leaf, a silicon chip without nanofibers was placed on top, without pressing, and left in place for 5 min. The treated leaf was then removed from the plant, the chip area was excised using a scalpel and the chip was removed. The leaf section was gently washed to remove surface FITC-dextran and imaged by confocal microscopy (a) Green channel, showing that epidermal cells evidenced no green (FITC) fluorescence (b) Red channel, showing autofluorescence from a few chloroplasts (c) Transmitted light channel, showing the structure of the tissue. (d) Merged image of (a), (b) and (c), showing that unimpaled cells do not take up FITC-dextran. (TIF) [file pone.0153621.s002.tif]

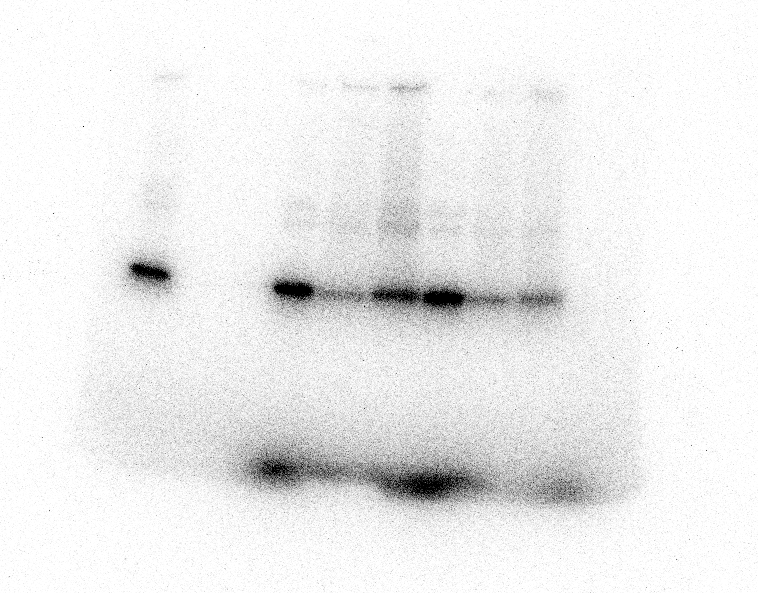

Supplement: S4 Fig — (TIF) [file pone.0153621.s004.tif]

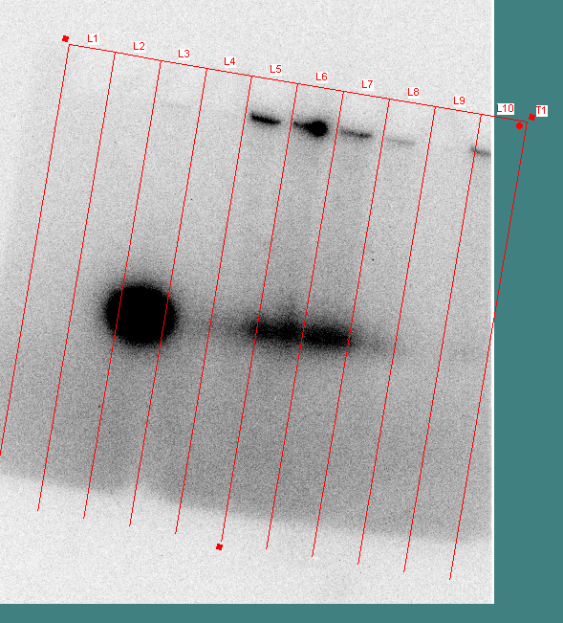

Supplement: S5 Fig — (TIF) [file pone.0153621.s005.tif]

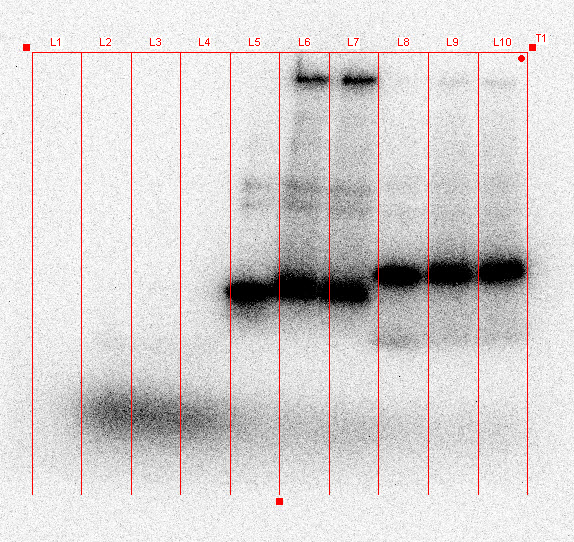

Supplement: S6 Fig — (TIF) [file pone.0153621.s006.tif]

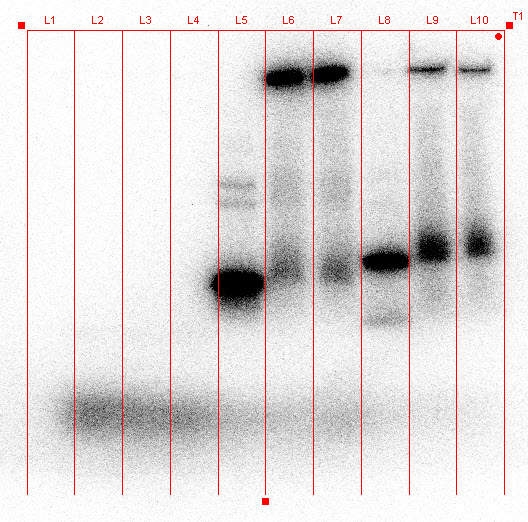

Supplement: S7 Fig — (TIF) [file pone.0153621.s007.tif]
